# Ionizing Radiation-induced Proteomic Oxidation in *Escherichia coli*

**DOI:** 10.1074/mcp.RA120.002092

**Published:** 2020-11-23

**Authors:** Steven T. Bruckbauer, Benjamin B. Minkoff, Deyang Yu, Vincent L. Cryns, Michael M. Cox, Michael R. Sussman

**Affiliations:** 1 Department of Biochemistry, University of Wisconsin-Madison, Madison, Wisconsin, USA; 2 Center for Genomic Science Innovation, University of Wisconsin School of Medicine and Public Health, Madison, Wisconsin, USA; 3 Department of Medicine, University of Wisconsin Carbone Cancer Center, School of Medicine and Public Health, University of Wisconsin-Madison, Wisconsin, USA

**Keywords:** Tandem mass spectrometry, oxidative stress, omics, bacteria, quantification, glyceraldehyde-3-phosphate dehydrogenase, ionizing radiation, proteome, protein oxidation

## Abstract

Recent work has begun to investigate the role of protein damage in cell death because of ionizing radiation (IR) exposure, but none have been performed on a proteome-wide basis, nor have they utilized MS (MS) to determine chemical identity of the amino acid side chain alteration. Here, we use *Escherichia coli* to perform the first MS analysis of IR-treated intact cells on a proteome scale. From quintuplicate IR-treated (1000 Gy) and untreated replicates, we successfully quantified 13,262 peptides mapping to 1938 unique proteins. Statistically significant, but low in magnitude (<2-fold), IR-induced changes in peptide abundance were observed in 12% of all peptides detected, although oxidative alterations were rare. Hydroxylation (+15.99 Da) was the most prevalent covalent adduct detected. In parallel with these studies on *E. coli*, identical experiments with the IR-resistant bacterium, *Deinococcus radiodurans,* revealed orders of magnitude less effect of IR on the proteome. In *E. coli*, the most significant target of IR by a wide margin was glyceraldehyde 3′-phosphate dehydrogenase (GAPDH), in which the thiol side chain of the catalytic Cys residue was oxidized to sulfonic acid. The same modification was detected in IR-treated human breast carcinoma cells. Sensitivity of GAPDH to reactive oxygen species (ROS) has been described previously in microbes and here, we present GAPDH as an immediate, primary target of IR-induced oxidation across all domains of life.

## Teaser

Measurement of ionizing radiation induced proteome-wide oxidative modification in both *E. coli* and the highly radiation resistant *D. radiodurans.* Although *E. coli* has several IR-induced oxidation events, *D. radiodurans* is virtually unaffected by IR treatment. An active site cysteine in the enzyme GAPDH within the glycolytic pathway was the largest IR-induced change in *E coli,* a result that replicated in human cells, suggesting GAPDH is partially responsible for IR response in higher organisms.

## Graphical Abstract

**Figure:**
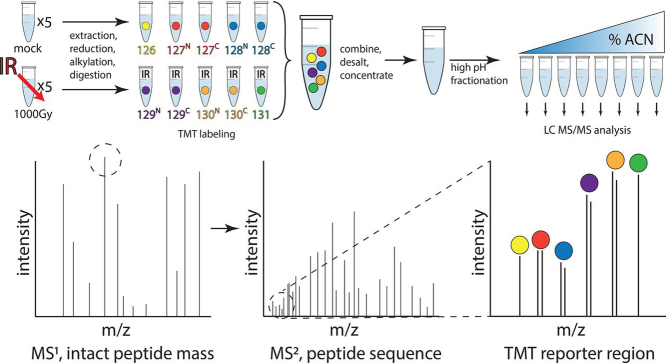

**Highlights**

• Quantifying ionizing radiation-induced oxidation in *E. coli* and *D. radiodurans.*
• Amelioration of protein damage dependent upon cellular components.
• GAPDH active site is highly modified and also IR-modified in *H. sapiens.*

Ionizing radiation (IR) is a ubiquitous source of lethal cellular damage to all organisms. The main source of such damage is reactive oxygen species (ROS) generated from IR-induced intracellular water radiolysis (1, 2, 3, 4). Hydrogen peroxide, superoxide, and hydroxyl radicals are the most prevalent types of ROS, and all can lead to oxidative modification of nucleic acids, proteins, and lipids (5).

Genetic and chemical strategies for survival after exposure to IR may involve a concerted response to minimize damage to essential metabolic machinery and where possible, to maximize its repair. DNA double-strand breaks (DSBs) accumulate at a rate of 3–6 DSBs/kGy/Mbp (6) and are lethal if not repaired by the appropriate enzymatic machinery. DNA damage is compounded by oxidative damage to proteins in the membrane and cytosol (6, 7, 8, 9). Increased membrane permeability caused by lipid and/or protein damage in the bilayer has been highlighted as a possible critical damaging event during irradiation (8).

The only known protein repair machinery is targeted to sulfur-containing residues (10, 11, 12, 13, 14, 15) and is incapable of repairing damage to the other 18 amino acids. Thus, although a significant number of proteins have evolved to repair DNA damage, there do not appear to be ROS degrading enzymes and protein repair machinery yet evolved which address oxidative damage to the proteome caused by the large scale generation of highly reactive and short lived (nanoseconds) hydroxyl radicals generated by IR (16).

The terrestrial background dose of IR (∼ 2.4 mGy/per year) is not sufficient to cause lethal damage. Thus, IR cannot act as a selective pressure in most organisms (1, 3). Indeed, the extremophile levels of IR resistance found in all domains of life has co-evolved with desiccation resistance (3, 6, 17). The bacterium *Deinococcus radiodurans* is the best-studied naturally IR-resistant species. *D. radiodurans* can survive IR doses more than 5000 Gy, orders of magnitude more than the lethal human dose (3, 5, 18, 19). This is likely because of the presence of both widely conserved and unique DNA repair enzymes and to the extraordinary ROS scavenging capacity of the *D. radiodurans* metabolome (3, 5, 6, 18, 19, 20, 21, 22).

Our current understanding of IR-induced damage to cellular macromolecules has been primarily based on *in vitro* studies, in which nucleotides, amino acids, peptides, or proteins have been exposed to IR in solution and the products characterized via MS (MS) (1, 23, 24, 25, 26, 27, 28, 29, 30). The few *in vivo* studies that detect and quantify IR-induced protein damage within intact cells have primarily relied on antibody-mediated detection of chemically derived adducts from initial oxidative modifications, primarily carbonylation (31, 32). This method is prone to false-positives and false negatives. Thus, with this methodology alone, one can miss many types of oxidation found on proteins (1, 33, 34) and be misdirected to others. Previous studies suggest that IR-induced oxidative damage to protein results in covalent modifications such as hydroxylation and carbonylation to amino acid side chains, as well as peptide bond hydrolysis (1, 23, 24, 25, 26, 27, 28, 29, 35). However, there have been no reports identifying the proteins that are the immediate *in vivo* targets of IR-mediated oxidation.

To identify and quantify IR-dependent oxidative modifications *in vivo*, we used modern MS methods and the model single-celled organism *Escherichia coli. E. coli* is an ideal subject for such comprehensive proteomic studies. Previous work has used MS to characterize the make-up of the *E. coli* proteome across several growth conditions (36, 37, 38, 39, 40, 41). As a direct comparison, we performed identical experiments using the IR-resistant microbe *D. radiodurans*. We quantify the extent of global oxidative modification to the *E. coli* and *D. radiodurans* proteomes caused directly by IR treatment and chemistries before any biological response.

## MATERIALS AND METHODS

##### Growth conditions and bacterial strains used in this study

Unless otherwise stated, *Escherichia coli* cultures were grown in Luria-Bertani (LB) broth at 37°C with aeration. *E. coli* were plated on 1.5% LB agar medium and incubated at 37°C. LB broth and agar was prepared as previously described (42). Overnight cultures were grown in a volume of 3 ml for 16 to 18 h. Overnight cultures were routinely diluted 1:100 in 10 ml of LB medium in a 50 ml Erlenmeyer flask and were grown at 37°C with shaking at 200 rpm and were harvested at an OD_600_ of 0.2. After growth to an OD_600_ of 0.2, cultures were placed on ice for 10 min to stop growth before being used for assays.

Where indicated, *E. coli* cells were grown in EZ defined rich medium (Teknova; Hollister, CA) supplemented with 0.2% glycerol. *Deinococcus radiodurans* was grown at 30°C in 2 × TGY broth (1% Tryptone, 0.6% Yeast Extract, 0.2% glucose per 1 L dH_2_0), or plated on 2 × TGY agar (with added 1.5% Bacto Agar per 1 L of media), as previously described (17). Plates were incubated until colonies were easily countable (48–72 h). Overnight cultures were incubated for 24 h before use. To generate exponential phase cultures for biological assays, overnight cultures were diluted 1:100 in 10 ml of 2 × TGY medium in a 50 ml Erlenmeyer flask and were grown at 30 °C with shaking at 200 rpm and were harvested at an OD_600_ of 0.08–0.15. Exponential phase cultures were placed on ice for 10 min to stop growth before being used for assays.

##### Ionizing radiation resistance assay to determine percent survival

Serial dilations, plating and irradiations were carried out as previously described (43). One ml sample for each dose tested (including 0 Gy) were removed and aliquoted into sterile 1.5 ml microfuge tubes. Samples were pelleted by centrifugation at 13 × *g* for 1 min, the supernatant removed, and resuspended in 1 ml ice-cold 1 × PBS (PBS). The pelleting process was repeated three more times to wash cells. A 100 μL aliquot of each culture was removed, serial diluted 1:10 in 900 μL of PBS to a final 10,000-fold dilution and 100 μL was plated on LB agar to determine the colony forming units (CFU)/ml before irradiation. Samples were maintained at 4°C and irradiated with the appropriate doses as described. After irradiation, a 100 μL aliquot of each culture was removed, diluted, and plated to determine CFU/ml and percent survival as described (43).

##### Western blot for protein carbonylation

Biological quintuplicate cultures of *E. coli* were grown overnight in LB from isolated colonies as described in *Growth Conditions*. Cultures were then diluted 1:100 in 25 ml LB in 125 ml Erlenmeyer flasks and grown to an OD_600_ of ∼0.2 as described. Two aliquots of 1 ml of each culture were then washed with 1× PBS and mock-treated or irradiated with 1000 Gy as described in *Ionizing Radiation Resistance Assay*. Following irradiation, 100 μL of each sample was removed and pelleted via centrifugation at 13 × *g* for 1 min Supernatants were removed, and pellets were resuspended in 12.5 μL of dH_2_O and 12.5 μL of 2× Laemilli sample buffer (250 mm Tris-HCl pH 6.8, 10% SDS, 20% glycerol, 10% β-Mercaptoethanol, sufficient Bromphenol Blue for dark blue coloration), boiled 5 min, and placed in 4°C. These samples were then used for the Oxyblot Protein Oxidation Detection Kit (Sigma Aldrich, St. Louis, MO; Cat# S7150) per manufacturer's protocols.

##### Mass spectrometry with E. coli after 10, 100, and 1000 Gy

A single culture of *E. coli* was grown overnight in LB from an isolated colony as described in *Growth conditions*. The culture was then diluted 1:100 in 10 ml LB in 3 separate 50 ml Erlenmeyer flasks and grown to an OD_600_ of ∼0.2 as described. Four ml of each culture was pelleted by centrifugation at 13 × *g* for 1 min per ml of culture (removing supernatant in between) and resuspended in 1 ml of 1× PBS. Each aliquot then washed with 1× PBS and irradiated with 10, 100, or 1,000 Gy as described in *Ionizing radiation resistance assay*. Before irradiation, 100 μμL was removed from each sample to assay CFU/ml of the untreated culture.

Samples were lysed by addition SDS to a final concentration of 2.0%, then immediately subjected to protein extraction and concentration using a standard methanol/chloroform protocol. Purified protein pellets were solubilized in 8M urea with 50 mm ammonium bicarbonate (ABC) and subjected to a standard BCA assay to determine protein concentration. Ten micrograms (varying volumes) of each was diluted to 4M urea with 50 mm ABC and treated with 2 mm DTT for 30 min at 50°C, 5 mm iodoacetamide for 30 min at room temperature in darkness, and then 2 mm DTT for 5 min at room temperature. Samples were diluted further to 1M urea with 50 mm ABC, and 0.05 μg of Trypsin and Lys-C proteases were each added (final protease mass/protein mass of 1:100). Samples were incubated overnight at 37°C, for 15 h total. Digestions were stopped with addition of neat formic acid to 1.0%, subjected to solid phase cleanup using Agilent C18 OMIX tips (Agilent Technologies; Santa Clara, CA), according to manufacturer's protocol, and dried down to completion using a vacuum centrifuge.

Samples were resolubilized into 0.1% formic acid and injected independently onto an Orbitrap Elite mass spectrometer (Thermo Fisher; Waltham, MA). For liquid chromatographic conditions, stationary phase was C18 and flow rate was 300nL/min. Mobile phases A and B were 0.1% formic acid in water and 0.1% formic acid in acetonitrile, respectively. For separation and elution, a 150-min gradient to 20% buffer B was used followed by a 12 min gradient to 50% B and 5 min gradient to 95%B.

Data-dependent acquisition was performed using a top-20 method with MS1 scans occurring at 120K resolving power in the Orbitrap and MS2 fragment ion scans occurring in the ion trap following CID fragmentation with a normalized collision energy of 35.0 units for +2 and greater charge states. Dynamic exclusion was enabled with a repeat count of 1 within a 30 s window.

Data were analyzed using the Sequest algorithm within Proteome Discoverer (PD) (Thermo Fisher). The Uniprot K12 *E. coli* proteome, downloaded on 7/2/2019 (PID: UP000000625, 4382 sequences including contaminants), was searched with the specified parameters: trypsin with 2 possible missed cleavages, precursor and fragment mass tolerance 10 ppm and 0.6 Da, respectively, and a max amount of 4 dynamic modifications per peptide. Dynamic modifications were specified as carbamidomethyl/+57.021 Da (on C), oxidation/+15.995 Da (on CDEFHILMNPQRSTVWY), carbonylation/+13.979 Da (on AEILQRSV), dioxidation/+31.990 Da (on CEFILMPRVWY), and trioxidation/+47.985 Da (on CFWY). Searches were based on previous reports of abundance of the given modifications on each amino acid reside (25). A false discovery rate (FDR) for peptide spectral matches (PSMs), peptides, and proteins of 0.05% was used via percolator in PD.

##### Preparation of wild-type E. coli and D. radiodurans samples for TMT mass spectrometry

*E. coli* or *D. radiodurans* cultures were grown overnight in biological quintuplicate and to an OD_600_ of 0.2 or 0.1 in EZ + 0.2% glycerol or 2× TGY, respectively in a total volume of 50 ml for each replicate. A 40 ml aliquot of each early exponential phase culture was pelleted by centrifugation at 3500 rpm for 10 min at 4°C. Supernatants were poured off, and samples resuspended in 40 ml of ice-cold 1× PBS. This process was repeated twice more with cells resuspended in 20 ml ice-cold PBS, and a final time suspending in 500 μL ice-cold 1× PBS. Four 100 μL aliquots were made for each culture in 1.5 ml microfuge tubes. Two for 0 and 1000 Gy for MS analysis, and two for plating to determine lethality.

##### TMT-labeled mass spectrometry with E. coli and D radiodurans

A cartoon illustration of the protocol is depicted in supplemental Fig. S2. Samples were lysed by addition SDS to a final concentration of 2.0%, then immediately subjected to protein extraction and concentration using a standard methanol/chloroform protocol. Purified protein pellets were solubilized in 8M urea with 50 mm triethylammonium bicarbonate (TEAB) and subjected to a standard BCA assay to determine protein concentration.

For each of the 10 samples, 10 μg (varying volumes) of each was diluted to 4M urea with 50 mm TEAB and treated with 2 mm DTT for 30 min at 50 °C, 5 mm iodoacetamide for 30 min at room temperature in darkness, and then 2 mm DTT for 5 min at room temperature. Samples were diluted further to 1M urea with TEAB, and 0.05 μg of Trypsin and Lys-C proteases were each added (final protease mass/protein mass of 1:100). Samples were incubated overnight at 37 °C, for 15 h total.

Digestions were stopped with addition of neat formic acid to 1.0%, subjected to solid phase cleanup using Agilent C18 OMIX tips (Agilent Technologies), according to manufacturer's protocol, and dried down to completion using a vacuum centrifuge. Dried samples were resolubilized into 50 mm TEAB. To each tube, 40 μg of each of the 10 tandem mass tag chemical adducts were added as follows (4:1 tag/protein): 126 - 0gy1; 127N - 0gy2; 127C - 0gy3; 128N - 0gy4; 128C - 0gy5; 129N - 1Kgy1; 129C - 1Kgy2; 130N - 1Kgy3; 13 °C - 1Kgy4; 131 - 1Kgy5.

Samples were incubated for 1 h at room temperature, after which 5% hydroxylamine was added to quench the reaction. Samples were immediately combined and purified/concentrated using a Waters 1 cc C18 Sep-pak solid phase chromatographic column (Waters Corporation; Milford, MA), according to manufacturer's protocol, then dried to completion with a vacuum centrifuge. Samples were resolubilized into 300 μL 0.1% formic acid for high-pH tip-based fractionation. Fractionation was carried out using a Pierce high pH reversed phase peptide fractionation spin column kit (Pierce Corporation; Junction City, OR), according to manufacturer's protocol. This resulted in nine fractions, which were dried down to completion using a vacuum centrifuge. Samples were resolubilized into 10 μL 0.1% formic acid for injection onto an Orbitrap Lumos mass spectrometer (Thermo Fisher). For liquid chromatographic conditions, stationary phase was C18 and flow rate was 275 nL/min. Three μL of each fraction was injected for analysis. Mobile phases A and B were 0.2% formic acid in water and 0.2% formic acid in 70% acetonitrile, respectively. For separation and elution, a 60 min gradient to 55% buffer B was used. DDA-MS was performed with the following parameters: MS1 spectra were acquired in profile mode in the Orbitrap with a resolution of 60K and a scan range of 350–1500 *m*/*z*. An AGC target of 1e6 and max inject time of 50 ms was used. Charge state filtering of 2–5, monoisotopic peak selection set to peptide, and dynamic exclusion of 60 s, *n* = 1 and with a mass tolerance of +/−25 ppm were used for triggering MS2 acquisition. Cycle time between MS1 scans was set to a max of 1 s. For MS2 acquisition, an isolation window of 1.6 Da was used, peptides were fragmented using HCD with a collision energy of 35%. MS2 were acquired in centroid mode in the orbitrap using the automatic scan range parameter with the first mass set to 100 and a resolution of 60K. An AGC target of 2e5 and a max inject time of 118 ms were used.

Data were analyzed using the Sequest algorithm within Proteome Discoverer (PD) (Thermo Fisher). For *E. coli* searches, the Uniprot K12 *E. coli* proteome, downloaded on 7/2/2019, was used (PID: UP000000625, 4382 sequences including contaminants). For *D. radiodurans* searches, the Uniprot *D. radiodurans* proteome, downloaded on 3/27/2018, was used (PID: UP000002524, 3172 sequences including contaminants) The respective databases were searched with the specified parameters: trypsin with 2 possible missed cleavages, precursor and fragment mass tolerance 10 ppm and 0.05 Da, respectively, and a max amount of 4 dynamic modifications per peptide. Dynamic modifications were specified as carbamidomethyl/+57.021 Da (on C), oxidation/+15.995 Da (on CDEFHILMNPQRSTVWY), carbonylation/+13.979 Da (on AEILQRSV), dioxidation/+31.990 Da (on CEFILMPRVWY), and trioxidation/+47.985 Da (on CFWY). Static modifications were specified as a TMT tag/+229.163 Da on N termini and K residues. Searches were based on previous reports of abundance of the given modifications on each amino acid reside (25). A false discovery rate (FDR) for peptide spectral matches (PSMs), peptides, and proteins of 0.05% was used via percolator in PD. For TMT quantification, tag abundances were normalized to the total tag/peptide amount per channel and a co-isolation filter was set for ≤30%. Peptide level data were exported, and further processing was performed in Excel as follows. An average of normalized abundance for the 1 kG channels over the 0 Gy channels was used to determine peptide level fold change, and two-tailed *t*-testing was used to calculate a *p* value per peptide. Adjusted *p* value were calculated using Benjamini-Hochberg correction.

##### Growth conditions and viability assays for mammalian cell lines used in this study

MDA-MB-231-mCherry cells were generated as previously described (44) and cultured in Invitrogen DMEM media (11695-092, Invitrogen) supplemented with 10% fetal bovine serum. Before irradiation, three plates of individually grown cells were harvested by trypsinization and washed with ice-cold PBS three times before splitting into two identical aliquots of 300,000 cells in 100 μl of PBS per set. Each aliquot was randomly assigned to be treated with either 0or 1000 Gy irradiation dosage. Following irradiation, 10,000 cells were seeded in duplicate in 96-well plate to assess survival using CellTiter-Glo® assay (Promega; Madison, WI; Cat #: G8461). CellTiter-Glo® assay was performed according the manufacturer's instruction following one, four, and 7 days following irradiation. Statistical significance was calculated using student's *t* test.

##### Label free quantification with MDA-MB-231 H. sapiens cell lines

The general experimental pipeline is depicted in supplemental Fig. S4. Cells in PBS were lysed, protein was extracted, and digested using S-trap micro columns according to manufacturer protocol (ProtiFi, LLC; Farmingdale, NY) and a combination of Trypsin and LysC. Digestions occurred overnight, and organic elutions were dried down to completion in a vacuum centrifuge and resolubilized into the aqueous elution volume. Peptide concentration was quantified using a micro-scale BCA assay. Ten micrograms of peptides per sample were desalted Agilent C18 OMIX tips (Agilent Technologies). Protocol was modified to include a 5% acetonitrile/0.1% formic acid wash step and peptides were eluted into 35% acetonitrile/0.1% formic acid, dried to completion using vacuum centrifugation, and resolubilized into 0.1% formic acid for MS analysis.

Samples were injected for analysis using onto an Orbitrap Lumos mass spectrometer (Thermo Fisher). Mobile phase A was 0.1% formic acid and mobile phase B was 80% acetonitrile/0.1% formic acid. A 70-min elution gradient to 37.5% B was used, after which 95% B was flushed for 5 min and column re-equilibration using 2% B was performed for 10 min We note that Orbitrap control software was updated to v3.3 between the acquisition of the *E. coli*/*D. radiodurans* data sets and the *H. sapiens* data set, and instrumental methods reflect this. DDA-MS was performed with the following parameters: MS1 spectra were acquired in profile mode in the Orbitrap with a resolution of 240K and a scan range of 350–2000 *m*/*z*. A normalized AGC target of 250% and automatic max inject time was used. Charge state filtering of 2–7, monoisotopic peak selection set to peptide, and dynamic exclusion of 45 s, *n* = 1 and with a mass tolerance of +/−10 ppm were used for triggering MS2 acquisition. Cycle time between MS1 scans was set to a max of 1s. For MS2 acquisition, an isolation window of 0.7 Da was used, peptides were fragmented using HCD with a collision energy of 28%. MS2 were acquired in centroid mode in the Ion Trap using the automatic scan range parameter and scan rate set to turbo. An AGC target of 3e4 and a max inject time of 25 ms were used.

Data were analyzed using the Sequest algorithm within Proteome Discoverer (PD) (Thermo Fisher). The Uniprot *H. Sapiens* from 6/9/2019 (PID: UP000005640, 20,353 sequences including contaminants), specifying once protein sequence per gene, was searched with the specified parameters: trypsin with 2 possible missed cleavages, precursor and fragment mass tolerance 10 ppm and 0.6 Da, respectively, and a max amount of 4 dynamic modifications per peptide. Dynamic modifications were specified as carbamidomethyl/+57.021 Da (on C), oxidation/+15.995 Da (on CDEFHILMNPQRSTVWY), carbonylation/+13.979 Da (on AEILQRSV), dioxidation/+31.990 Da (on CEFILMPRVWY), and trioxidation/+47.985 Da (on CFWY). No static modifications were set. Searches were based on previous reports of abundance of the given modifications on each amino acid reside (25). A false discovery rate (FDR) for peptide spectral matches (PSMs), peptides, and proteins of 0.05% was used via percolator in PD. For quantification, a combination of the Minora Feature Detector, Feature Mapper, and Precursor Ions Quantifier nodes were used in PD. Default settings were used for the Minora Feature Detector and Feature Mapper nodes. For the Precursor Ion Quantifier node, Intensity was used for precursor quantification, normalization was performed using total peptide amount per file, and adjusted *p* value were used. Peptide and modified peptide level quantification was used for calculation.

##### Preparation and irradiation of whole cell, cell lysate, and pure protein E. coli samples

A cartoon illustration of the protocol is depicted in supplemental Fig. S5. Grew quintuplicate overnight cultures of *E. coli* MG1655 as described in *Growth conditions*. Fifty ml LB medium was inoculated with 500 μL of overnight culture and grown to an OD_600_ of 0.2 as described. Forty ml of cultures were aliquoted into separate 50 ml conical tubes and were pelleted by centrifugation at 5000 rpm, 4°C, for 10 min Supernatant was removed and cells were resuspended in 10 ml ice cold 1 × PBS. Pelleting and resuspension was repeated three more times, however final resuspension was done in 800 μL PBS. Made six 100 μL aliquots of each culture into separate 1.5 ml tubes. Placed each on ice. For lysate and pure protein prep at 0 and 1,000 Gy, froze four 100 μL aliquots for each sample at –80 °C. This is the first of two freeze/thaw cycles to lyse cells.

Two of the whole cell samples were kept on ice (two for each replicate cultures) and irradiated as described with a dose of 1000 Gy; the other replicate received no dose. Samples that received no dose and 1000 Gy were returned to laboratory and frozen at –80 °C. After 2 h, we removed all samples from −80 °C and thawed at room temperature for 10 min all samples were frozen overnight at −80 °C.

The following day, we removed and thawed the lysate samples at room temperature for 10 min These samples were irradiated as described for the whole cell samples. Post irradiation, we removed and thawed the whole cell samples at room temperature for 10 min All whole cell and cell lysate samples were pelleted via centrifugation at 13 × *g* for 2 min one-hundred μL aliquots of supernatant for each sample was added to 3500 MWCO Slide-a-lyzer Mini-Dialysis Units (Thermo Fisher; Cat #69550). Mini-Dialysis Units were floated on 500 ml 1× PBS with very slow stirring with stir bar for 2 h at room temperature. The dialysis was repeated twice, overnight for the final repeat.

The next day, to set-aside an aliquot of purified protein for each sample to run on an SDS-PAGE gel, 25 μL of each sample was removed and added to 25 μL of 2× Laemilli sample buffer (250 mm Tris-HCl pH 6.8, 10% SDS, 20% glycerol, 10% β-Mercaptoethanol, sufficient Bromphenol Blue for dark blue coloration), boiled 5 min, and placed in 4°C. The remaining whole cell and cell lysate samples from dialysis were moved into fresh 1.5 ml tubes, flash-frozen with liquid N_2_, and stored at –80 °C. The pure protein samples were removed and thawed at room temperature for 10 min. The steps for pelleting and dialyzing samples were repeated as described above for the whole cell and lysate samples. Because dialyzed protein ends up with greater than 100 μL, the volumes were split into two tubes as close to 100 μL as possible. Samples were irradiated as described above. After irradiation, the appropriate samples were re-combined in a single 1.5 ml tube. As described above, 25 μL aliquots were removed and stored at 4°C to run on an SDS-PAGE gel. Remaining pure protein samples from dialysis were placed in fresh 1.5 ml tubes and flash-frozen with liquid N_2_, and stored at –80°C before TMT-MS analysis, without pre-fractionation.

Fourteen μl of each sample was loaded and run on a Bio-Rad pre-cast 12% SDS-PAGE protein gel (Bio-Rad, Hercules, CA; 10 well Product #: 4561043; 15 well Product #: 4561046). Gels were run at 100 V until dye from Laemmli sample buffer had run off the gel. Gel was then stained per manufacturer's protocol using the Silver Stain Plus kit (Bio-Rad; Cat #: 1610449).

##### Experimental design and statistical rationale

This study contains 2 major and 2 minor proteomic experiments. The first minor proteomic experiment was performed before the majority of the work described herein and was used to measure both lethality and oxidative state of *E. coli* over a dosage of treatments. Given the 99.9% lethality we observed in combination with significant oxidation, we chose a 1000 Gy dosage point for our larger scale studies.

The major 2 experiments, which we consider to be the major focus here, were performed using *E. coli* and *D. radiodurans.* For each of these, biological quintuplicate cultures were grown and used for either ionizing radiation- or mock-treatment, considered control samples. Each of these replicates was labeled using a single channel from the TMT 10-plex kit (Thermo Fisher), and the 10 channels for *E. coli* and *D. radiodurans* fractionated and analyzed as described above. Eight *E. coli* fractions were analyzed, and nine *D. radiodurans* fractions were analyzed. Quintuplicates were chosen because of amenability to TMT 10-plex labeling and quantification. Post-normalization across channels to an even peptide load per channel in Proteome Discoverer, average abundance calculations, and Benjamini-Hochberg correction of *p* value were performed in Excel given the data set size, large *n* per samples, and a desire for stringent significance thresholds. This was performed in Excel so that randomization of data could be performed and processed identically as the output data set. The randomization shown was performed using the AbleBits add-on to excel and abundances were shuffled between all 10 channels per peptide at random as this was deemed to be the most appropriate to maintain relative magnitudes of abundance per peptide while randomizing for true biological effects of IR treatment.

The final proteomic experiment detailed herein was with 231 *H. sapiens* cells. In this experiment, biological duplicates were mock- or IR-treated LFQ in the absence of fractionation were used (experimental design is depicted in supplemental Fig. S4). This experiment was treated as a survey for IR response of *H. sapiens* GAPDH response, to ask whether the active site cysteine was similarly oxidized by IR treatment. For this reason, calculation of abundance ratios and Benjamini-Hochberg correction of *p*-values was performed using Proteome Discoverer. No randomization was performed with this data set.

## RESULTS

##### Quantification of IR-induced modification to the E. coli and D. radiodurans proteomes

Although much previous work has examined biological responses to IR exposure, we have chosen to focus on the immediate, abiotic chemical effects of IR on the *in vivo* proteome before any biological response. To ensure our observations of IR-induced changes to the *E. coli* proteome are indeed abiotic, cultures were cooled to 4°C before irradiation and extensively washed in 1× PBS (PBS) to halt metabolism and remove nutrients in growth media that may act as a radioprotectant. In addition, sample tubes were submerged in cold (4–10°C) water throughout irradiation. In this study, IR was delivered at 70 Gy/min by a high energy electron beam linear accelerator (Linac) commonly used in cancer radiotherapy. Based upon preliminary experiments using IR dosages of 0, 10, 100, and 1000 Gy to *E. coli,* we chose to move forward with and examine in depth the effects at specifically 1000 Gy in more detail (supplemental Table S1). Under the conditions used in our experiments, a dose of 1000 Gy was administered quickly (∼15 min) and killed 99.9% of a population of *E. coli* MG1655, as quantified by colony counts of pre- and post-irradiation cultures (supplemental Fig. S1A). Based on previous measurements, this dose is sufficient to cause approximately 20 DNA double-strand breaks (DSBs) per cell and is also expected to induce significant oxidative damage to proteins (6), a result which we observed as well (supplemental Fig. S2). To provide an IR-resistant proteome control, we carried out an identical experiment as laid out above with the highly radioresistant bacterium *D. radiodurans*. The ability of this bacterium to protect its proteome from IR-generated ROS is a well-studied phenomenon (6, 18, 20, 31). At a dose of 1000 Gy, this bacterium exhibits no lethality (supplemental Fig. S1A).

To survey and quantify as much IR-induced oxidation as possible, peptides from ten replicates (five treated and five mock-treated) were labeled using the tandem mass tag (TMT) 10-plex labeling kit. These were combined and subjected to high-pH fractionation, and fractions were analyzed using MS (MS). Database searches on a whole proteome level focused on previously identified IR-induced amino acid modifications, including carbonylation (+13.98 Da), hydroxylation (+15.99 Da), dioxidation (+31.99 Da), and trioxidation (+47.99 Da) (25, 29). We note here that although we classify +31.99 Da as dioxidation, such a mass shift could also be peroxidation. Though these modifications represent different chemistry, we cannot distinguish between the two in these data. We also note that we are using general terms for modification here, though more specific terminology will be used in some instances (*i.e.* +47.99 Da on Cys results in a sulfonic acid chemical group, +13.98 Da on Pro can represent conversion to pyroglutamic acid, etc.). We additionally attempted to identify several previously reported, less-common modifications (25, 29, 30); however, none of these appeared to any significant degree in our data sets.

Overall, we quantified 13,262 peptides from *E. coli*, corresponding to 1938 unique proteins. This set of proteins represents approximately half of the encoded *E. coli* proteome based on predicted open reading frames, or nearly two-thirds of proteins expressed during exponential-phase growth in rich medium (39). Of the quantified peptides, 11,703 exhibited no statistically significant change in abundance after IR treatment. For the subset of peptides that showed no change in abundance, single hydroxylation (+15.99 Da) was the most prevalent modification (10.8%) (Table I). We take this subset of modified peptides to represent background modification in our whole cell samples and is consistent with previous observations of endogenous ROS produced during growth in nutrient-rich medium (45, 46) and oxidation of proteins during the electrospray ionization step of MS (47).

**Table I:** Numbers of detected and quantified peptides in irradiated and unirradiated samples of *Escherichia coli* determined by mass spectrometry

*Escherichia coli* MS data	Total detected	Total quantified	No fold change (irradiated/control)	Fold increase >1 (irradiated/control)	Fold increase >2 (irradiated/control)	Fold decrease <1 (irradiated/control)	Fold decrease <0.5 (irradiated/control)
Total peptides	15108	13262	11703	764	48	795	2
Carbonylated peptides (+13.979 Da)	117	80	72	5	0	3	0
Hydroxylated peptides (+15.99 Da)	1861	1664	1437	175	19	52	0
Dioxidized peptides (+31.99 Da)	351	285	247	24	2	14	0
Trioxidized peptides (+47.985 Da)	55	41	28	11	2	2	0
Percent carbonylated	0.8%	0.6%	0.6%	0.7%	0.0%	0.4%	0.0%
Percent hydroxylated	12.3%	12.5%	12.3%	22.9%	39.6%	6.5%	0.0%
Percent dioxidized	2.3%	2.1%	2.1%	3.1%	4.2%	1.8%	0.0%
Percent trioxidized	0.4%	0.3%	0.2%	1.4%	4.2%	0.3%	0.0%

*Significance of fold changes in abundance were determined with a threshold of Benjamini-Hochberg adj. *p*-value < 0.05.

The other 1559 peptides exhibited a statistically significant increase or decrease in abundance (adjusted *p*-value < 0.05) (Table I). Of the 764 peptides with IR-induced increased abundance (fold change > 1), 175 were hydroxylated, by far the most prevalent adduct. There were 24 instances of dioxidation, the second-most frequent modification (Table I). Increased abundance of such modified was taken to be a direct chemical result of IR treatment. Of modified peptides with IR-induced increases in abundance, methionine sulfoxide was by far the most significantly induced modification (137 instances), followed by dihydroxytryptophan (20 instances) and cysteine sulfonic acid (8 instances) (Table II). Within the set of 795 peptides with decreased abundance (fold change <1), modifications were less prevalent. Only 52 were hydroxylated and 14 were dioxidized.

**Table II:** Numbers of detected and quantified peptides in irradiated and unirradiated samples of *Deinococcus radiodurans* determined by mass spectrometry

*Deinococcus radiodurans* MS data	Total detected	Total quantified	No fold change (irradiated/control)	Fold increase >1 (irradiated/control)	Fold increase >2 (irradiated/control)	Fold decrease <1 (irradiated/control)	Fold decrease <0.5 (irradiated/control)
Total peptides	13777	11526	11525	1	1	0	0
Carbonylated peptides (+13.979 Da)	189	134	134	0	0	0	0
Oxidized peptides (+15.99 Da)	2198	1779	1778	1	1	0	0
Dioxidized peptides (+31.99 Da)	498	399	399	0	0	0	0
Trioxidized peptides (+47.985 Da)	43	26	26	0	0	0	0
Percent carbonylated	1.4%	1.2%	1.2%	0.0%	0.0%	N/A	N/A
Percent hydroxylated	16.0%	15.4%	15.4%	100.0%	100.0%	N/A	N/A
Percent dioxidized	3.6%	3.5%	3.5%	0.0%	0.0%	N/A	N/A
Percent trioxidized	0.3%	0.2%	0.2%	0.0%	0.0%	N/A	N/A

*Significance of fold changes in abundance were determined with a threshold of Benjamini-Hochberg adj. *p*-value < 0.05.

Most peptides that exhibited statistically significant changes in abundance after IR exposure were unmodified. We hypothesize that both increases and decreases in this subset are caused by ROS-induced peptide bond cleavage. In the case of decreases, we propose that protein-level fragmentation affects regions of proteins such that less of a given proteotypic peptide is ultimately present in the sample. Conversely, in some cases, ROS-induced cleavage facilitates proteolytic digestion and thus production of proteotypic peptides, resulting in increased abundance for a pool of unmodified peptides post-IR treatment. These hypotheses have not yet been tested. Overall, a 1000 Gy dose of IR had an immediate, measurable effect on the *E. coli* proteome, with hydroxylation being the predominant observable modification.

**Fig. 1:**
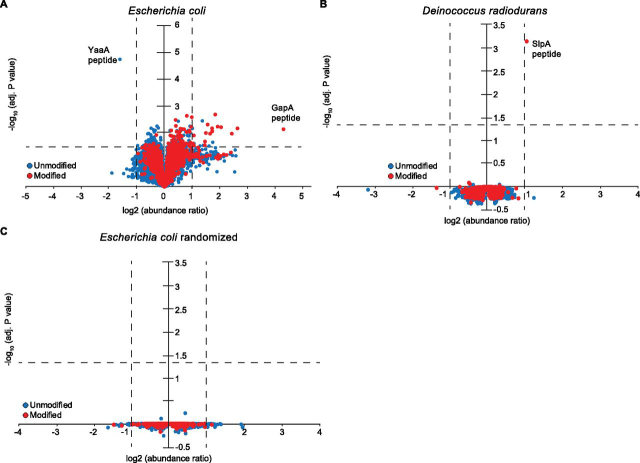
**Increase in abundance of oxidized peptides because of IR exposure.***A*, Of the nearly 13,996 quantified peptides (indicated by each circle) in *E. coli* cells, approximately 12% have statistically significant (fold change greater or less than 1, Benjamini-Hochberg adj. *p*-value < 0.05) changes in abundance because of 1000 Gy of irradiation. *B*, When *D. radiodurans* is irradiated with 1000 Gy under identical conditions as *E. coli*, there is only one peptide out of the 11,715 that shows a significant fold change in abundance. *C*, When the *E. coli* data set is randomized across the ten channels (five without treatment, five with), and fold changes are recalculated, there are no statistically significant changes observed. The spread of fold changes with low significance closely resembles the *D. radiodurans* data set.

To benchmark our observations with *E. coli*, we carried out an identical experiment using the highly radioresistant bacterium *D. radiodurans*, irradiated with 1000 Gy or mock-treated. Prevention of IR-induced oxidation to the proteome in *D. radiodurans* is a well-studied phenomenon (6, 18, 20, 31, 32). Additionally, previous studies have used MS to characterize the *D. radiodurans* proteome identifying up to 1400 proteins under given growth and stress conditions (48, 49). However, no work has yet assayed IR-induced oxidation on a large proteomic scale. Thus, using the same workflow described above for *E. coli*, we identified 1815 *D. radiodurans* proteins (∼58% of the genome-encoded proteome) from 11,526 quantified peptides (Table III). As was the case in the *E. coli* samples, there was a background of oxidatively modified peptides with no significant change in abundance. Approximately 14% of the quantified peptides were hydroxylated (+15.99 Da) which, similar to *E. coli*, was the most prevalent modification (Table III). However, the *D. radiodurans* proteome was markedly less affected by IR than that of *E. coli*. Only a single hydroxylated peptide was observed with a significant IR-induced increase in abundance (adjusted *p*-value < 0.05) (Fig. 1*B*). This single peptide maps to an abundant, extracellular protein of the slime layer of *D. radiodurans*, SlpA (50). Therefore, in stark contrast to *E. coli*, *D. radiodurans* does not exhibit a measurable increase in oxidative modifications to the proteome after exposure to IR.

**Table III:** Amino acid modifications on peptides with significant increases in abundance because of IR treatment

Residue	Carbonylation (+13.979 Da)	Hydroxylation (+15.99 Da)	Dioxidation (+31.99 Da)	Trioxidation (+47.985 Da)
A	2	N/A	N/A	N/A
C	N/A	0	0	8
D	N/A	1	N/A	N/A
E	1	1	0	N/A
F	N/A	4	0	0
G	N/A	N/A	N/A	N/A
H	N/A	1	N/A	N/A
I	1	1	0	N/A
K	N/A	0	N/A	N/A
L	2	6	0	N/A
M	N/A	137	0	N/A
N	N/A	0	N/A	N/A
P	N/A	0	N/A	N/A
Q	0	0	N/A	N/A
R	0	0	0	N/A
S	0	3	N/A	N/A
T	N/A	0	N/A	N/A
V	2	1	0	N/A
W	N/A	6	20	3
Y	N/A	4	2	0
Total	8	165	22	11

*N/A indicates that the residue was not searched for that modification, as detailed in Methods.

There are two potential sources of the high background of oxidatively modified peptides that do not change in abundance because of IR exposure, endogenous intracellular ROS species or source oxidation during electrospray ionization. The observed background is similar in both *E. coli* and *D. radiodurans. D. radiodurans* has a documented enhanced capacity to ameliorate ROS (31, 32). If we assume that *D. radiodurans* is better able to suppress intracellular ROS than is *E. coli*, yet the background of oxidatively modified peptides is similar or even higher than in *E. coli*, the likely origin of our oxidized peptide background is in-source oxidation during electrospray ionization of peptides. Determining the precise source of these IR-independent modifications is outside the focus of this study.

To validate the observed “shotgun-like” effect of numerous, small fold changes in peptide abundance as a direct result of IR treatment of *E. coli*, we randomized the abundance values between the 10 TMT channels for each individual peptide quantified and recalculated the fold changes and adjusted *p*-values for the quintuplicate control and IR-treated samples. As shown in Fig. 1*C*, there were zero peptides that met the criteria of adjusted *p*-value of <0.05 in the randomized data set, lending confidence to the large number of IR-induced modifications that were small in magnitude but nonetheless statistically significant.

##### What factors determine which proteins are targeted by IR?

Our data set indicates that IR induces abiotic modification to the *E. coli* proteome. However, there are ∼4000 predicted proteins in the *E. coli* proteome. We next asked whether any factors determine which proteins are affected by IR-induced oxidation. In a cellular environment, proteins are surrounded by hydration spheres of coordinated water molecules (51, 52). As IR-induced damage to cellular macromolecules is caused primarily by reactive oxygen species (ROS) formed by radiolysis of water, the surface area (size of the hydration sphere) of a given protein may significantly affect the vulnerability of that protein to IR-induced damage. The abundance of a given protein would further increase the target size. Therefore, we hypothesized that simple target theory, which states that a larger, more abundant protein provides a more likely target, might dictate whether a protein is susceptible to IR-induced damage. To test the target theory hypothesis, we developed the relative absolute mass (RAM) metric, which incorporates both the mass (a general predictor of protein surface area) and abundance per protein. For abundance values, we used the most comprehensive protein abundance catalogue of *E. coli* to date (40).

**Fig. 2:**
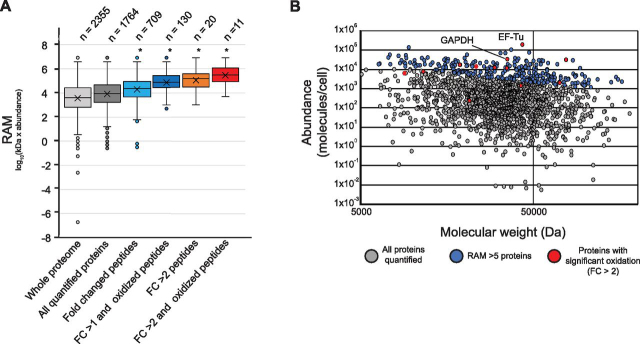
**Proteins with greater molecular weight and abundance are more prone to IR-induced hydroxylation.***A*, Relative absolute mass (RAM) values of proteins detected. A RAM value was calculated using abundance and molecular weight values from *E. coli* grown in medium with glycerol as the sole carbon source, as previously reported (40). RAM was used as a rough predictor of target size of each protein in *E. coli*. The distribution RAM values for all proteins detected in the Schmidt *et al.* study (40) are pictured in light gray, and those that were detected in this study are pictured in dark gray. The distribution of RAM values for all proteins mapped from peptides with significant fold changes (fold change in abundance >1 or <1, Benjamini-Hochberg adj. *p*-value <0.05.) is pictured in light blue, and the hydroxylated peptides with the same significance criteria is pictured in dark blue. The distribution of RAM values for all proteins mapped from peptides with fold changes in abundance >2 or <0.5 (Benjamini-Hochberg adj. *p*-value <0.05.) is pictured in orange, and the hydroxylated peptides with the same significance criteria is pictured in red. “*n*” values indicate the number of proteins that fall into each category. The “*” symbol indicates that the distributions are significantly different with an adjusted *p*-value > 1 × 10^−5^ as calculated by a 2-tailed Student's *t* test. *B*, Separated molecular weight and abundance values of proteins detected. Each protein detected was graphed as circle based on the molecular weight and abundance values utilized to calculate the RAM metric (40). Glyceraldehyde 3′-phosphate dehydrogenase (GAPDH; encoded by *gapA*) and elongation factor Tu (Ef-Tu; encoded by *tufA*) are indicated.

The peptides that change in abundance with IR treatment, regardless of modification state, generally map to proteins with a significantly higher average RAM (4.3) than the average RAM of the whole proteome data set (3.9) (Fig. 2). The average RAM of proteins containing modified peptides with increased abundance greater than 1 or 2 is ∼4.9 and 5.4, respectively. However, 256 proteins have a RAM value >5 and we detect oxidative modifications on only 57 such proteins (with a fold increase greater than 1). Thus, although target theory may loosely approximate which proteins are initial targets of IR-induced damage, it does not fully explain the observed chemical effects of IR on the proteome, particularly why some peptides are far more susceptible to IR than others.

Nearly 10% of all peptides detected showed a statistically significant change in abundance at a threshold of greater or less than 1. Setting a stricter threshold for consideration of a 2-fold change up or down, which suggests greater susceptibility to IR, revealed only 48 peptides significantly increase in abundance, and only 2 peptides decrease in abundance (Fig. 1*A*). Twenty-two of the 48 peptides with increased abundance were oxidatively modified at least once; no peptides with decreased abundance were modified. (Table IV). Thus, of the 13,262 peptides quantified in *E. coli*, only 22 had an oxidative modification induced by IR greater than 2-fold. Most of these peptides exhibited hydroxylation; only a single increased peptide had a covalent carbonylation. Of these significantly induced modifications, methionine was the most susceptible amino acid (7 confirmed methionine sulfoxide residues) followed by leucine (5 hydroxyleucine modifications) (Table IV).

**Table IV:** Hydroxylated peptides with significantly increased abundance because of IR exposure in E. coli

Protein	Master protein descriptions	Protein accession	Relative protein abundance	Peptide sequence	Modifications	Fold change (treated: untreated)	Adjusted *p*-value	Manual validati on of oxidative modification
GapA	Glyceraldehyde-3-phosphate dehydrogenase A	P0A9B2	0.170	[K].YAGQDIVSNASCTTNCLAPLAK.[V]	1xTrioxidation [C12]; 1 ×TMT6plex [K22]; 1 ×TMT6plex [N-Term]; 1 ×Carbamidomethyl [C16]	20.12	0.005	OK
TalB	Transaldolase B	P0A870	0.059	[R].LTIAPALLK.[E]	1xHydroxylation [L8]; 1 ×TMT6plex [K9]; 1 ×TMT6plex [N-Term]	3.16	0.008	OK
Pal	Peptidoglycan-associated lipoprotein	P0A912	0.085	[K].MYLQGK.[G]	1xHydroxylation [M1]; 1 ×TMT6plex [K6]; 1 ×TMT6plex [N-Term]	5.35	0.043	OK
				[R].S.D.FAQMLDAHANFLR.[S]	1xHydroxylation [M6]; 1 ×TMT6plex [N-Term]	2.41	0.038	OK
LptE	LPS-assembly lipoprotein	P0ADC1	0.001	[R].SFFDNPQMALAK.[D]	1xHydroxylation [M8]; 1 ×TMT6plex [K12]; 1 ×TMT6plex [N-Term]	2.98	0.003	OK
HtpG	Chaperone protein	P0A6Z3	0.010	[R].LTDTPAIVSTDADEMSTQMAK.[L]	2xHydroxylation [M15; M19]; 1 ×TMT6plex [K21]; 1 ×TMT6plex [N-Term]	2.10	0.009	OK
RplB	50S ribosomal protein L2	P60422	0.064	[K].HPVTPWGVQTK.[G]	1xHydroxylation [P/W]; 1 ×TMT6plex [K11]; 1 ×TMT6plex [N-Term]	2.77	0.022	Ambiguous, likely W6
RpmB	50S ribosomal protein L28	P0A7M2	0.032	[R].FWVESEK.[R]	1xTrioxidation [W2]; 1 ×TMT6plex [K7]; 1 ×TMT6plex [N-Term]	2.22	0.049	OK
RpsD	30S ribosomal protein S4	P0A7V8	0.070	[R].MGFGATR.[A]	1xHydroxylation [M1]; 1 ×TMT6plex [N-Term]	2.03	0.049	OK
RpsN	30S ribosomal protein S14	P0AG59	0.040	[R].EAAMRGEIPGLK.[K]	1xHydroxylation [M4]; 1 ×TMT6plex [K12]; 1 ×TMT6plex [N-Term]	2.85	0.007	OK
				[K].AIISDVNASDEDRWNAVLK.[L]	1xDioxidation [W14]; 1 ×TMT6plex [K19]; 1 ×TMT6plex [N-Term]	2.40	0.035	OK
FusA	Elongation factor G	P0A6M8	0.163	[K].IATDPFVGNLTFFR.[V]	1xHydroxylation [F6]; 1 ×TMT6plex [N-Term]	3.33	0.005	OK
TufA	Elongation factor Tu	P0CE47	1.000	[R].AFDQIDNAPEEK.[A]	1xHydroxylation [F2]; 1 ×TMT6plex [K12]; 1 ×TMT6plex [N-Term]	6.30	0.005	OK
				[K].VGEEVEIVGIK.[E]	1xHydroxylation [I10]; 1 ×TMT6plex [K11]; 1 ×TMT6plex [N-Term]	5.46	0.008	OK
				[R].AGENVGVLLR.[G]	1xHydroxylation [L8]; 1 ×TMT6plex [N-Term]	4.17	0.004	OK
				[K].STCTGVEMFR.[K]	1xDioxidation [E7]; 2 ×Hydroxylation [M8; F9]; 1 ×TMT6plex [N-Term]; 1 ×Carbamidomethyl [C3]	3.82	0.004	Ambiguous, likely either dioxidation on E7, or dioxidation on M8, or single hydroxylation on M8 and F9
				[R].HTPFFK.[G]	1xHydroxylation [H1]; 1 ×TMT6plex [K6]; 1 ×TMT6plex [N-Term]	3.60	0.001	OK
				[K].LLDEGR.[A]	1xHydroxylation [L1]; 1 ×TMT6plex [N-Term]	3.34	0.006	OK
				[R].QVGVPYIIVFLNK.[C]	1xHydroxylation [L11]; 1 ×TMT6plex [K13]; 1 ×TMT6plex [N-Term]	2.78	0.016	OK
				[R].TVGAGVVAK.[V]	1xHydroxylation [V7]; 1 ×TMT6plex [K9]; 1 ×TMT6plex [N-Term]	2.10	0.023	OK
				[K].TTLTAAITTVLAK.[T]	1xHydroxylation [I/L]; 1 ×TMT6plex [K13]; 1 ×TMT6plex [N-Term]	2.05	0.019	Ambiguous
YgfK	Putative oxidoreductase	Q46811	N/A	[K].SLGFGVK.[L]	2xHydroxylation [L2; F4]; 1 ×TMT6plex [K7]; 1 ×TMT6plex [N-Term]	2.34	0.027	OK

*Peptides listed are those with a significant fold increase (fold change >2, Benjamini- Hochberg adj. *p*-value <0.05.) and have at least one modification. If the oxidative modification was localized to a residue, the residue number in the peptide is listed and the residue is underlined. If our analysis could not determine which residue was modified, the candidate residues are separated by “/” symbols. Each oxidative modification was additionally assessed by manual inspection of mass spectra, as indicated by the far-right column. Protein accession numbers are from UniProt (85). Relative abundance values are based on previously reported values (40) and are those used for RAM analysis; being the most abundant *E. coli* protein, EF-Tu is set to “1.” YgfK abundance was not previously quantified, and therefore does not have a relative abundance value.

**Fig. 3:**
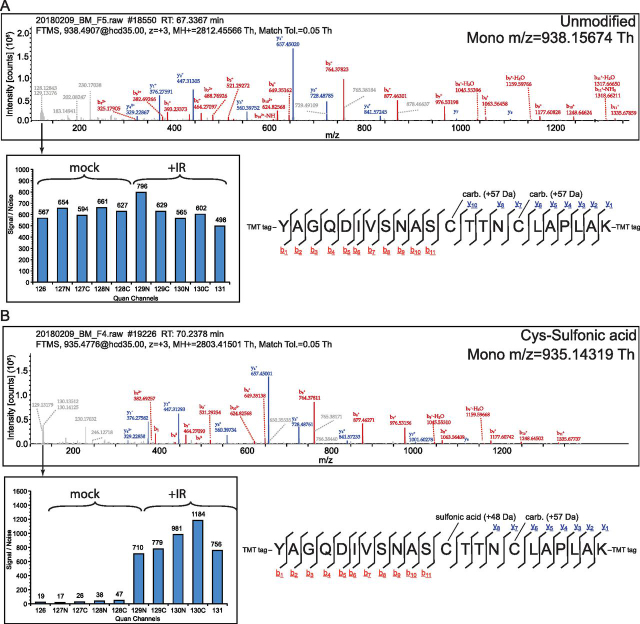
**Mass spectra of *E. coli* GAPDH active site peptides.***A*, Example fragment ion spectra of (*A*) nonoxidized and *B*, sulfonic acid-modified active site cysteine of *E. coli* GAPDH. Sequence and fragment ion diagram correspond to annotated spectra and identified ions in the y- and b- series are underlined in respective colors.

Two proteins were the clearest targets of IR-induced damage: elongation factor Tu (Ef-Tu) and glyceraldehyde 3′-phosphate dehydrogenase A (abbreviated GapA in *E. coli*, or GADPH). Nine unique peptides with an oxidative modification induced greater than 2-fold map to Ef-Tu, the most of any observed protein. Given Ef-Tu is the most abundant protein in *E. coli* by nearly a factor of two (40) and these peptides vary in location in Ef-Tu and in terms of fold increase, these modifications appear to represent the “shotgun-like” effect of IR induced ROS on a highly abundant protein. In stark contrast, a single peptide containing a mass shift consistent with a trioxidation event (+47.99 Da) in place of a Cys carbamidomethylation mapping to the catalytic Cys within the active site of GAPDH increased in abundance by nearly 20-fold, the most of any single peptide detected by a large margin (Table IV, Fig. 3).

##### The active site of GAPDH is the primary target of IR

The unique sensitivity of the catalytic Cys residue of GAPDH to oxidative modifications from H_2_O_2_ (53, 54, 55, 56, 57, 58, 59) and reactive nitrogen species (RNS) such as peroxynitrite and nitric oxide (60, 61, 62, 63, 64) has been characterized. Much of this previous literature has focused on oxidation of the Cys side chain to sulfenic acid, which can be further glutathionylated or form a disulfide with the nearby Cys residue and be repaired by glutaredoxins or thioredoxins (10, 65, 66). Recently, the catalytic Cys has been implicated as a redox thiol switch responsible for altering cellular metabolism under oxidative stress conditions (57, 58, 67, 68, 69). However, GAPDH has not previously been identified as a major target of IR-induced ROS *in vivo*.

IR irreversibly modified the catalytic Cys of GAPDH to Cys sulfonic acid (Fig. 3**)**. This modified active site peptide was increased approximately 20-fold in abundance, by far the greatest increase of any peptide quantified **(**Fig. 1*A***)**. Furthermore, we observed an IR-induced sulfonic acid modification in our preliminary experiment with *E. coli* treated with 1000 Gy, but not at doses of 100 or 10 Gy (supplemental Data set S5). There are multiple mechanisms whereby the Cys thiol group can be converted to sulfonic acid through sequential oxidation events (25, 30). We do not know which of these has led to this modification here, as the method measures reaction endpoints. We additionally detected both the unmodified and singly hydroxylated (on a flanking threonine) forms of this active site peptide which did not change in abundance with IR treatment. Twenty-two other peptides mapping to GAPDH were quantified; however, only one other peptide (which was unmodified) exhibited a greater than 2-fold increase in abundance (Table V). Overall, there are 3 Cys residues in the *E. coli* GAPDH. Of those, we only identified the sulfonic acid conversion on the catalytic Cys noted above. In the entire data set, we observed 1348 peptides containing Cys residues. Of these, 40 peptides (including the GAPDH active site) had an annotated Cys sulfonic acid conversion. Of those 26, the GAPDH active site was the only event to exhibit an IR-dependent, statistically significant increase. This IR-induced sulfonic acid thus appears to be highly specific to the active site GAPDH.

**Table V:** All GAPDH peptides quantified in this study

Protein	Peptide sequence	Modifications	Fold change (treated:untreated)	Adjusted *p*-value	Manual localization of oxidative modification
*Escherichia coli* GAPDH (Accession number: P0A9B2)	[K].YAGQDIVSNASCTTNCLAPLAK.[V]	1xCarbamidomethyl [C16]; 1 ×Trioxidation [C12]; 1 ×TMT6plex [K22]; 1 ×TMT6plex [N-Term]	20.12	5.1E-03	OK
	[K].AGIALNDNFVK.[L]	1xTMT6plex [K11]; 1 ×TMT6plex [N-Term]	2.02	4.7E-03	N/A
	[K].LTGMAFR.[V]	1xOxidation [M4]; 1 ×TMT6plex [N-Term]	1.97	4.1E-03	OK
	[K].DNTPMFVK.[G]	1xTMT6plex [K8]; 1 ×TMT6plex [N-Term]	1.92	7.3E-03	N/A
	[K].LTGMAFR.[V]	1xTMT6plex [N-Term]	1.90	3.9E-03	N/A
	[K].DNTPMFVK.[G]	1xOxidation [M5]; 1 ×TMT6plex [K8]; 1 ×TMT6plex [N-Term]	1.88	5.8E-03	OK
	[R].GASQNIIPSSTGAAK.[A]	1xTMT6plex [K15]; 1 ×TMT6plex [N-Term]	1.86	9.5E-03	N/A
	[R].FDGTVEVK.[D]	1xTMT6plex [K8]; 1 ×TMT6plex [N-Term]	1.86	1.9E-02	N/A
	[K].LVSWYDNETGYSNK.[V]	1xTMT6plex [K14]; 1 ×TMT6plex [N-Term]	1.83	6.6E-03	N/A
	[K].VGINGFGR.[I]	1xTMT6plex [N-Term]	1.79	9.1E-03	N/A
	[K].VLPELNGK.[L]	1xTMT6plex [K8]; 1 ×TMT6plex [N-Term]	1.71	6.6E-03	N/A
	[R].VPTPNVSVVDLTVR.[L]	1xTMT6plex [N-Term]	1.52	5.7E-03	N/A
	[K].AATYEQIK.[A]	1xTMT6plex [K8]; 1 ×TMT6plex [N-Term]	1.52	2.2E-02	N/A
	[K].VVMTGPSK.[D]	1xTMT6plex [K8]; 1 ×TMT6plex [N-Term]	1.49	2.7E-02	N/A
	[R].VPTPNVSVVDLTVRLEK.[A]	1xTMT6plex [K17]; 1 ×TMT6plex [N-Term]	1.43	2.8E-01	N/A
	[K].VVMTGPSK.[D]	1xOxidation [M3]; 1 ×TMT6plex [K8]; 1 ×TMT6plex [N-Term]	1.39	2.5E-02	OK
	[K].AAAEGEMK.[G]	1xOxidation [M7]; 1 ×TMT6plex [K8]; 1 ×TMT6plex [N-Term]	1.33	1.4E-01	OK
	[R].VTAERDPANLK.[W]	1xTMT6plex [K11]; 1 ×TMT6plex [N-Term]	1.32	1.8E-01	N/A
	[K].GANFDK.[Y]	1xTMT6plex [K6]; 1 ×TMT6plex [N-Term]	1.30	2.4E-01	N/A
	[K].VLDLIAHISK.[-]	1xTMT6plex [K10]; 1 ×TMT6plex [N-Term]	1.28	1.4E-01	N/A
	[K].AAAEGEMK.[G]	1xTMT6plex [K8]; 1 ×TMT6plex [N-Term]	1.21	7.9E-02	N/A
	[K].YAGQDIVSNASCTTNCLAPLAK.[V]	2xCarbamidomethyl [C12; C16]; 1 ×TMT6plex [K22]; 1 ×TMT6plex [N-Term]	1.12	3.6E-01	N/A
	[K].YAGQDIVSNASCTTNCLAPLAK.[V]	2xCarbamidomethyl [C12; C16]; 1 ×Oxidation [T13]; 1 ×TMT6plex [K22]; 1 ×TMT6plex [N-Term]	1.06	6.5E-01	Ambiguous between T13/T14
	[K].DGHLIVNGK.[K]	1xTMT6plex [K9]; 1 ×TMT6plex [N-Term]	1.03	9.3E-01	N/A
	[K].TVDGPSHK.[D]	1xTMT6plex [K8]; 1 ×TMT6plex [N-Term]	0.90	7.8E-01	N/A
*Deinoccous radiodurans* GAPDH (Accession number: Q9RUP1)	[R].GVEVVAINDLTDNHTLAHLLK.[Y]	1xTMT6plex [K21]; 1 ×TMT6plex [N-Term]	1.21	1.7E + 00	N/A
	[K].KVIITAPAK.[G]	2xTMT6plex [K1; K9]; 1 ×TMT6plex [N-Term]	1.19	1.1E + 00	N/A
	[R].VLDLPHSDLRR.[A]	1xTMT6plex [N-Term]	1.11	1.2E + 00	N/A
	[R].IADLVQLVQNK.[G]	1xTMT6plex [K11]; 1 ×TMT6plex [N-Term]	1.10	1.1E + 00	N/A
	[K].VIDEAFGIEK.[A]	1xTMT6plex [K10]; 1 ×TMT6plex [N-Term]	1.09	1.3E + 00	N/A
	[R].AAAINIIPTSTGAAK.[A]	1xTMT6plex [K15]; 1 ×TMT6plex [N-Term]	1.08	1.5E + 00	N/A
	[R].VPTPTGSISDVSVILGR.[D]	1xTMT6plex [N-Term]	1.07	1.2E + 00	N/A
	[R].VLDLPHSDLR.[R]	1xTMT6plex [N-Term]	1.07	1.3E + 00	N/A
	[K].AIMTTVHSYTNDQR.[V]	1xTMT6plex [N-Term]	1.07	1.0E + 00	N/A
	[K].AVSQVYPALK.[G]	1xOxidation [Y6]; 1 ×TMT6plex [K10]; 1 ×TMT6plex [N-Term]	1.07	1.2E + 00	OK
	[K].AVSQVYPALK.[G]	1xOxidation [Y6]; 1 ×Carbonyl [V5]; 1 ×TMT6plex [K10]; 1 ×TMT6plex [N-Term]	1.06	1.0E + 00	OK
	[K].AIMTTVHSYTNDQR.[V]	2xOxidation [M3; N/I]; 1 ×TMT6plex [N-Term]	1.04	1.0E + 00	Yes M3, Ambiguous on second between Y9-N11
	[K].IQAIAERDPANIK.[W]	1xTMT6plex [K13]; 1 ×TMT6plex [N-Term]	1.02	1.0E + 00	N/A
	[R].DVTVEEVNNVFR.[E]	1xTMT6plex [N-Term]	1.02	1.0E + 00	N/A
	[K].AVSQVYPALK.[G]	1xTMT6plex [K10]; 1 ×TMT6plex [N-Term]	1.01	1.0E + 00	N/A
	[K].AVSQVYPALK.[G]	1xDioxidation [Y/P]; 1 ×TMT6plex [K10]; 1 ×TMT6plex [N-Term]	0.99	1.0E + 00	Ambiguous, almost certainly on Y6
	[R].FDGTVEYDESSLTVNGK.[K]	1xTMT6plex [K17]; 1 ×TMT6plex [N-Term]	0.95	1.0E + 00	N/A
	[K].AIMTTVHSYTNDQR.[V]	1xOxidation [T/V/M]; 1 ×TMT6plex [N-Term]	0.92	1.1E + 00	Ambiguous, almost certainly on M3
	[K].FDGTSLR.[V]	1xTMT6plex [N-Term]	0.90	1.0E + 00	N/A
	[K].VIITAPAK.[G]	1xTMT6plex [K8]; 1 ×TMT6plex [N-Term]	0.89	1.3E + 00	N/A
	[K].IQAIAER.[D]	1xTMT6plex [N-Term]	0.87	1.2E + 00	N/A
*Homosapiens* GAPDH (Accession number: P04406)	[R].VPTANVSVVDLTCR.[L]		2.116	1.41E-04	N/A
	[K].VIHDNFGIVEGLMTTVHAITATQK.[T]	1xOxidation [M/T/L]	1.694	1.39E-03	Ambiguous, almost certainly on M13
	[R].VVDLMAHMASK.[E]		1.464	1.15E-02	N/A
	[R].VPTANVSVVDLTCR.[L]	1xCarbamidomethyl [C13]	1.452	5.22E-03	N/A
	[R].VVDLMAHMASKE.[-]		1.405	3.38E-02	N/A
	[K].LISWYDNEFGYSNR.[V]	1xDioxidation [W/Y]; 2 ×Oxidation [W4; Y5]	1.271	2.54E-01	Ambiguous, almost certainly Dioxidation on W4
	[K].LTGMAFR.[V]		1.259	1.88E-01	N/A
	[K].LVINGNPITIFQERDPSK.[I]		1.176	6.23E-01	N/A
	[K].VGVNGFGR.[I]		1.161	5.83E-01	N/A
	[R].VIISAPSADAPMFVMGVNHEK.[Y]		1.158	5.95E-01	N/A
	[R].VVDLMAHMASKE.[-]	1xOxidation [L/H/M]	1.126	9.27E-01	Ambiguous, almost certainly on M5
	[K].IISNASCTTNCLAPLAK.[V]	2xCarbamidomethyl [C7; C11]	1.122	7.72E-01	N/A
	[K].VIPELNGK.[L]		1.107	8.35E-01	N/A
	[K].RVIISAPSADAPMFVMGVNHEK.[Y]		1.069	9.89E-01	N/A
	[R].GALQNIIPASTGAAK.[A]		1.021	1.00E + 00	N/A
	[K].LVINGNPITIFQER.[D]		0.98	1.00E + 00	N/A
	[R].VVDLMAHMASK.[E]	1xOxidation [M/H]	0.961	1.00E + 00	Ambiguous, almost certainly on M5
	[K].LISWYDNEFGYSNR.[V]	1xCarbonyl [E8]	0.935	9.89E-01	OK
	[K].LISWYDNEFGYSNR.[V]	1xOxidation [W4]	0.882	9.28E-01	OK
	[K].WGDAGAEYVVESTGVFTTMEK.[A]	1xCarbonyl [E7]	0.879	9.45E-01	OK
	[K].AGAHLQGGAK.[R]		0.865	9.27E-01	N/A
	[R].VIISAPSADAPMFVMGVNHEK.[Y]	1xDioxidation [F13]; 2 ×Oxidation [M12; M15]	0.851	6.68E-01	Ambiguous, most likely hydroxylation on both M12/M15
	[K].LISWYDNEFGYSNR.[V]		0.844	6.29E-01	N/A
	[K].LISWYDNEFGYSNR.[V]	1xTrioxidation [Y/W]	0.841	9.72E-01	Ambiguous between W4/Y5
	[K].WGDAGAEYVVESTGVFTTMEK.[A]		0.811	3.95E-01	N/A
	[K].QASEGPLK.[G]		0.803	3.43E-01	N/A
	[K].WGDAGAEYVVESTGVFTTMEK.[A]	1xOxidation [M/Y/E/S]	0.702	2.10E-02	Ambiguous, almost certainly on M19
	[R].VIISAPSADAPMFVMGVNHEK.[Y]	1xOxidation [M/F/P]	0.694	1.57E-02	OK
	[K].TVDGPSGK.[L]		0.617	7.53E-03	N/A
	[K].VIHDNFGIVEGLMTTVHAITATQK.[T]		0.491	1.30E-09	N/A
	[K].WGDAGAEYVVESTGVFTTMEK.[A]	1xOxidation [T18]; 1 ×Carbonyl [E7]	0.491	1.77E-01	Yes E7, Ambiguous hydroxylation on T18 or M19
	[R].VVDLMAHMASK.[E]	2xOxidation [M5; M8]	0.474	1.14E-04	OK
	[K].GILGYTEHQVVSSDFNSDTHSSTFDAGAGIALNDHFVK.[L]		0.281	6.56E-16	N/A
	[K].IISNASCTTNCLAPLAK.[V]	1xCarbamidomethyl [C]; 1 ×Dioxidation [C7]; 2 ×Oxidation [S3; S6]	1000 Gy only	6.56E-16	Ambiguous between hydroxylation on S3/S6 and dioxidation on C7
	[K].IISNASCTTNCLAPLAK.[V]	1xCarbamidomethyl [C11]; 1 ×Trioxidation [C7]	1000 Gy only	6.56E-16	OK


*Peptides listed map to the GAPDH protein of each respective organism. Adjusted p-values listed are Benjamini-Hochberg adjusted. Each oxidative modification was assessed by manual inspection of mass spectra, as indicated by the far-right column. Protein accession numbers are from UniProt (85).

The active site motif of GAPDH (SCTTNC; the first Cys being the catalytic residue) is nearly invariant among eukaryotes and bacteria (supplemental Fig. S3) (57, 70, 71, 72). Thus, GAPDH may be a target of IR across kingdoms of life. We note that the *D. radiodurans* GAPDH active site peptide lacks the nearby Lys residue seen in *E. coli* GAPDH, and was therefore neither identified nor quantified using tryptic digestion and MS analysis. We successfully quantified 21 other peptides from *D. radiodurans*, none of which changed in abundance or modification because of IR exposure (Table V). Intriguingly, the *Deinococcus-Thermus* phylum of bacteria, which includes *D. radiodurans*, lacks the second Cys residue of the SCTTNC active site conserved from *E. coli* to *Homosapiens* (supplemental Fig. S3).

**Fig. 4:**
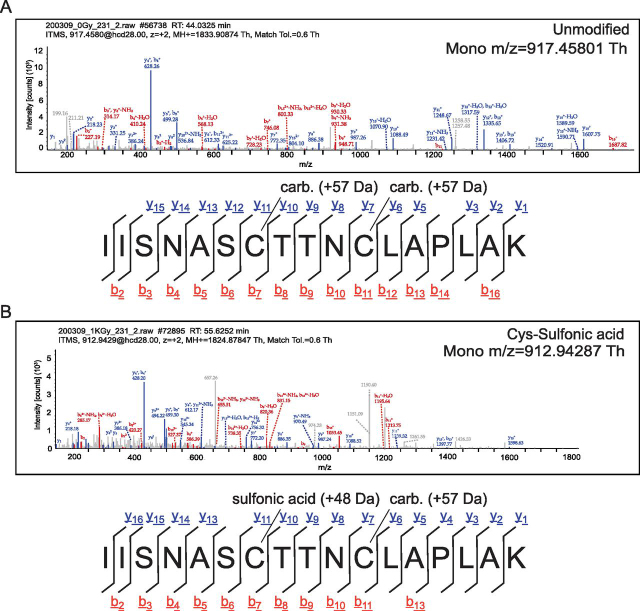
**Mass spectra of *H. sapiens* GAPDH active site peptides.***A*, Example fragment ion spectra of (*A*) nonoxidized and *B*, sulfonic acid-modified active site cysteine of *H. sapiens* GAPDH from the endothelial breast carcinoma cell line MDA-MB-231. Sequence and fragment ion diagram correspond to annotated spectra and identified ions in the y- and b- series are underlined in respective colors.

To determine whether the GAPDH active site Cys is sensitive to IR outside of *E. coli*, we performed identical irradiation treatment using the human breast carcinoma cell line MDA-MB-231 (abbreviated 231). We again irradiated with 1000 Gy, which was sufficient to induce 99% lethality 1 day post-exposure (supplemental Fig. S1B). The main target of this exploratory experiment was to probe the active site Cys of *Homosapiens* GAPDH for oxidative modification–to do this, we used biological duplicates of 231 cells and a performed a label free quantification (LFQ)-MS experiment. Full quantification of the extent of IR-induced damage to the human proteome, which is ∼5-fold larger than the *E. coli* proteome, will be the subject of future work. However, we quantified 35 peptides mapping to GAPDH in either the irradiated or unirradiated 231 cells. Of those, two heavily-oxidized peptides mapping to the active site appeared only in the irradiated replicates. The first contained the same IR-induced conversion of the catalytic Cys residue to Cys sulfonic acid (Fig. 4), and the second contained a +31.99 Da mass shift to sulfinic acid or possibly oxidation of nearby Ser residues, though it is more likely, given the respective chemistries, that the modification falls on the Cys (Table V). Thus, we propose that irreversible oxidation of GAPDH is a conserved consequence of extreme IR exposure across domains of life.

**Fig. 5:**
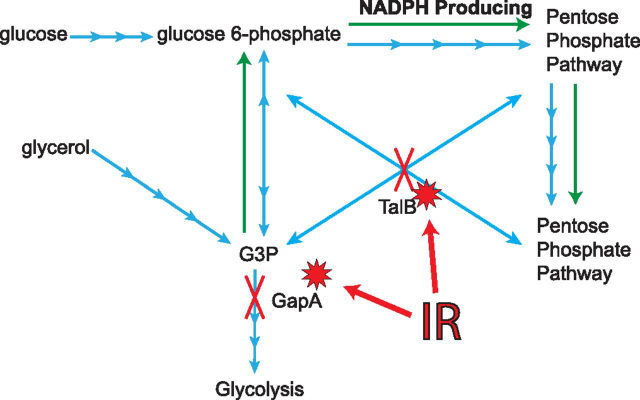
**Graphical summary of significantly hydroxylated proteins detected in this study.** Abbreviations are as follows: ionizing radiation (IR), NADPH (NADPH), glyceraldehyde 3′-phosphate (G3P). Hydroxylation events are indicated by a red starburst. Blue arrows are indicative of enzymatic reactions in glucose and glycerol metabolism. Green arrows indicate the directionality of flux through these pathways in response to hydroxylation of TalB and GapA.

Inactivating oxidation of the GAPDH active site shifts carbon flux from glycolysis to the pentose-phosphate pathway to generate NADPH for use in reduction reactions necessary to combat protein oxidation (10). In addition to oxidation of GAPDH, among the peptides which have a greater than 2-fold IR-induced modification is a peptide mapping to the *E. coli* transaldolase TalB (Table IV). This protein funnels glycolytic intermediates into late stages of the pentose phosphate pathway that do not produce NADPH. Oxidation of GAPDH and TalB in concert likely further enhances carbon flux through the NADPH-production reactions of the pentose phosphate pathway (Fig. 5). GSH is an intracellular antioxidant involved in ROS amelioration and is present in all domains of life (73, 74). Abundant NADPH is required to maintain reduced GSH pools. In *E. coli*, GSH is the second-most abundant intracellular metabolite (75), and therefore may play a role in amelioration of IR-induced ROS.

**Fig. 6:**
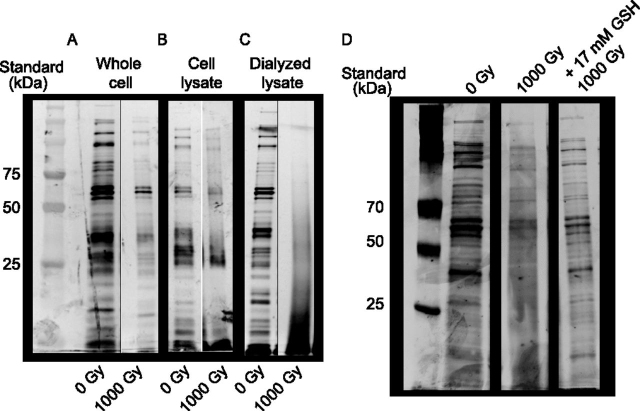
**The *E. coli* proteome is protected from IR-induced degradation by low-molecular weight species present in the cell lysate.***A*, Protein from irradiated and unirradiated whole cells, *B*, cell lysates, and *C*, dialyzed lysates were prepared and observed as described in the *Materials and methods* and as illustrated in supplemental Fig. S5D, Sensitivity of dialyzed cell lysates with added GSH. A physiologically relevant concentration of GSH (GSH), 17 mm, (75) was added to dialyzed cell lysates before irradiation as described in the Materials and Methods section.

To investigate this, we first asked whether endogenous small molecules play a role in preventing IR-induced proteome damage. Using SDS-PAGE, we found that lysing cells before IR treatment resulted in easily observable IR-induced proteome degradation. To add to this, dialyzing away small molecules following lysis before irradiation led to significantly more IR-induced degradation (Fig. 6). We hypothesized that this degradation was mediated entirely by IR-produced ROS, and indeed, adding a physiologically relevant concentration of GSH to the dialyzed proteome sample before IR treatment prevented observable degradation entirely (Fig. 6). These results collectively suggest that low molecular weight reductants such as GSH, and the ability to maintain an abundant pool of these metabolites through inactivation of GAPDH and increases in NADPH production, may be crucial for a cell to survive ROS stress because of IR exposure.

## DISCUSSION

There are five major conclusions of this work. *First*, ionizing radiation (IR)-inflicted oxidative damage to the *Escherichia coli* proteome by 1000 Gy was widespread but modest, affecting about 10% of the identified proteome. Most of that damage occurred at quite low levels, involving increases or decreases of <2-fold in peptide abundance. Only a small percentage of the peptides with altered abundance were modified by IR (Fig. 1). *Second*, the IR-induced changes, although modest, were substantially greater than those seen in the highly IR-resistant bacterium *Deinococcus radiodurans*, which exhibited only a single instance of an IR-induced modification when subjected to IR at 1000 Gy (Fig. 1). *Third*, immediately following Linac-generated IR treatment, the most common oxidative modification was hydroxylation (+15.99 Da mass shift) rather than carbonylation (+13.98 Da), dioxidation/peroxidation (+31.99 Da) or trioxidation (+47.99 Da) (Table I). *Fourth*, whereas *E. coli* does not protect its proteome to the extent observed in *D. radiodurans*, a substantial level of protection is evident, nonetheless. This was reflected both in our large-scale MS (MS) experiments (Fig. 1) as well as proteome degradation assays (Fig. 6). *Fifth,* and finally, the oxidative effects of IR on the proteome are not entirely random. The active site Cys residue in glyceraldehyde 3'-phosphate dehydrogenase (GAPDH) is a major and highly specific target of oxidation by IR.

We have successfully begun to catalog the extent of chemical damage caused by IR to the *E. coli* proteome before any biological response, *i.e.* the “abiotic response.” To that end, we have directly quantified one of the largest subsets of the expressed *E. coli* proteome yet reported in each growth condition (1938 unique protein identifications from 13,262 quantified peptides). Nearly 10% of all quantified peptides significantly changed in abundance because of IR exposure (adjusted *p*-value < 0.05) (Table I; Fig. 1*A*). This “shotgun-like” effect is particularly striking when comparing the observed values to that calculated from a randomized *E. coli* data set (Fig. 1*C*). However, most peptides with altered abundance featured small fold changes. Only 22 modified peptides increased greater than 2-fold because of IR exposure in *E. coli*. Of these, hydroxylation (+15.99 Da; primarily on Met and Leu) was the most prevalent modification. No carbonylations were detected at this significance threshold. Our results strongly suggest that protein hydroxylation, rather than carbonylation, is the most important abiotic and immediate proteomic consequence of cellular exposure to IR.

The lack of IR-induced damage to the *D. radiodurans* proteome (Table III; Fig. 1*B*) is consistent with previous studies (6, 31, 32). In fact, the pattern of IR-induced changes to peptide abundance with this microorganism closely resembles that of the randomized *E. coli* data set (Fig. 1). Only one peptide was significantly hydroxylated (+15.99 Da) because of IR exposure in *D. radiodurans*. This single event is on an abundant protein found on the outer slime layer, SlpA. SlpA has been previously implicated in resistance to environmental stress (50, 76). In addition to our effort to characterize oxidation of the *D. radiodurans* proteome, we note that our data set includes the greatest number of proteins yet detected in *D. radiodurans* in a single growth condition (11,526 quantified peptides corresponding to over 1800 proteins) (48, 49, 77).

Whereas prokaryotic systems generally contain their genetic material within a single DNA molecule, the ranges of protein concentration and three-dimensional shape and size within a proteome can vary immensely. We thus used the product of copy number and molecular weight to assign a Relative Absolute Mass (RAM) value to each protein we identified. Target theory, the classical explanation for damage accumulation to DNA, predicts that the proteins with the highest RAM values are the most likely to be modified in response to IR. The most striking example of this is the strong effect of IR on the oxidation of elongation factor Tu (Ef-Tu). Ef-Tu has the highest RAM value in *E. coli* (6.9), is the most abundant cellular protein by nearly a factor of two (40), and 8 peptides were oxidatively modified in response to IR throughout the protein. In contrast, there are many high RAM proteins that were not modified suggesting that target theory alone cannot predict the obtained results.

Underscoring the idea that target theory does not wholly predict IR-sensitivity of proteins, the most striking result from our data were irreversible oxidation of the buried active site of GAPDH. A single peptide mapping to the active site of GAPDH revealed IR-induced modification of the catalytic Cys (Cys151) to Cys sulfonic acid; this modification was by far the most strongly IR-induced oxidation event in the entire data set (a 20-fold increase in abundance). Target theory does not predict such extreme and specific targeting of GAPDH, suggesting that irreversible oxidation of Cys151 may have a biologically significant role downstream of this initial oxidation event. The unusual reactivity of this buried catalytic Cys residue with H_2_O_2_ and reactive nitrogen species (RNS) has been extensively documented (53, 54, 55, 56, 57, 58, 59, 60, 61, 62, 63, 64, 65, 67, 78, 79, 80, 81). However, this study provides the first evidence that GAPDH may be the primary protein target of the oxidizing environment created by acute irradiation. Specifically, catalytic inactivation via irreversible modification of Cys151 to Cys sulfonic acid (Fig. 3) within the active site may be an initial step in the subsequent biological response to ROS stress and DNA damage (53, 57).

The active site motif (SCTTNC) of GAPDH is nearly ubiquitous across bacteria and eukaryotes (supplemental Fig. S3). The exception is the second Cys residue, (Cys155 in *E. coli*) which is not necessary for normal catalytic function of GAPDH, and is typically buried in a hydrophobic region near the active site. It is, however, necessary for sensitizing Cys151 to oxidation by H_2_O_2_, leading to the sulfenylation, sulfonylation, and GSH-dependent glutathionylation reported in earlier work (57, 65, 78, 80, 81). This hypothesis is strengthened and underscored by results from a previous study demonstrating that mutation from the highly conserved SCTTNC motif to the *D. radiodurans* sequence SCTTNS decreases GAPDH sensitivity to H_2_O_2_ (57). In the present study, we also determined that the GAPDH of *D. radiodurans* exhibits no detectable oxidation at the active site. We acknowledge that the overall capacity of Deinococcus to ameliorate reactive oxygen species and protect its proteome (3, 18, 20, 31, 32) may explain this result in whole or in part. However, the C to S mutation might also reflect a lack of evolutionary pressure to maintain such a sensitive oxidation sensor in IR resistant organisms. In fact, the domains in which the SCTTNC active site sequence is not maintained (particularly in the Kingdom Archaea and the Phylum *Deinococcus-Thermus* which includes *D. radiodurans* (supplemental Fig. S3) (57) may suggest novel stress responses in these organisms that do not require GAPDH to maintain their oxidative stress sensing capability. The full implications of the evolutionary discrepancy between GAPDH active site sequences remain to be explored.

We further sought to confirm that GAPDH is a target of IR in a distantly related, eukaryotic organism. When irradiated with the same dose as the *E. coli* cells used herein, the human breast carcinoma cell line MDA-MB-231 exhibits the same IR-induced sulfonic acid modification on the catalytic Cys of GAPDH (Fig. 4). Like the *E. coli* results, most of the 35 quantified GAPDH peptides only exhibited small (less than 2-fold) changes in abundance. However, in addition to the sulfonylated side chain of the catalytic Cys, we also detected IR-induced sulfinic acid modification (+31.99 Da mass shift) (Table V).

We note that although commonly reported oxidative modifications to the GAPDH catalytic Cys are reversible (*i.e.* sulfenic acid, disulfide formation, glutathionylation), the IR-induced sulfonic acid which we detect is not. Although functional characterization of the IR-induced sulfonylated GAPDH remains to be carried out, previous studies have shown that this modification irreversibly inactivates the enzyme (53, 56, 57, 58, 60, 67, 82). Inactivation of GAPDH by oxidative stress shifts carbon flux to the pentose phosphate pathway as a means of bolstering the NADPH pool for use in reduction reactions (56, 57, 58, 78), including GSH reduction. We now report that this phenomenon likely occurs after exposure to IR across domains of life and precedes the biological response to such extreme stress.

The primary target for IR-mediated modification stands alone and does not conform to target theory. This is a modified peptide from glyderaldehyde-3-phosphate dehydrogenase (GAPDH; encoded by *gapA*), which exhibited a nearly 20-fold increase in cells exposed to 1000 Gy (Table IV). This single peptide includes the highly conserved GAPDH active site motif, SCTTNC (Fig. 3). Within the active site, the thiol side chain of the catalytic cysteine residue (C151) was irreversibly oxidized to sulfonic acid in the *E. coli* data set. This oxidative event appears highly targeted, as only two other peptides in *E. coli* GAPDH were modified (very modestly in comparison) by IR (Table V). Despite evolutionary distance, the same Cys modification was induced by IR in the epithelial breast carcinoma cell line MDA-MB-231 when irradiated with the same dose of IR (Fig. 4).

We thus propose that GAPDH is the most significant protein target of IR in *E. coli,* a target that is also prominent in *H. sapiens* cells. This strong, IR-dependent modification on the strictly conserved active site motif suggests that the role GAPDH plays as an oxidative stress sensor is both ancient and important enough to be maintained across evolutionary time scales. The result also indicates that the active site structure of GAPDH has evolved to facilitate this Cys oxidation event. Such an important role in exposure to IR adds to the growing list of cellular functions of GAPDH (53, 66, 83, 84). Taken in total, the data presented herein suggest that GAPDH is a key player in the response to IR exposure, from Bacteria to Eukarya.

## Data Availability

All raw data are freely available via the Chorus Project (Project ID# 1606). All processed .pdresult files are freely available via scientific data repository Zenodo.org at https://zenodo.org/record/3834859.
