# Supplementary material for: Ionizing Radiation-induced Proteomic Oxidation in Escherichia coli
Source: Mol Cell Proteomics. 2020 Nov 23;19(8):1375–95. doi: 10.1074/mcp.RA120.002092 (PMC8015010; doi:10.1074/mcp.RA120.002092)
Supplement: Supplementary file 1 [file mmc1.zip › 160164_2_supp_545970_qbpy2f.pdf]

Supplemental figures

Figure S1. Percent survival of relevant organisms after exposure to 1,000 Gy. A) Percent survival of *E. coli* and *D. radiodurans*. Exponential phase cell cultures were exposed to 1,000 Gy of high-energy electron beam irradiation as described in the *Materials and Methods* section. Percent survival was calculated via determining colony-forming units (CFU)/mL of cultures with and without irradiation. Each irradiation was performed in biological triplicate. **B)** Survival of human epithelial breast carcinoma cell line MDA-MD-231 after a dose of 1,000 Gy. Cell culture and viability tests were conducted as described in the *Materials and methods*.

Figure S2. Detecting protein oxidation induced by IR via western blot. A) Biological quintuplicate *E. coli* cultures were mock-treated or treated with 1,000 Gy of IR as described in the *Materials and Methods* section. After irradiation, cells were lysed and a portion of the supernatants were **A)** run on an SDS-PAGE gel and visualized via silverstain or **B)** used for the Oxyblot Protein Oxidation Detection Kit as directed by manufacturer.

Figure S3. Conservation of the active site motif of GAPDH. The amino acid resides with IR-induced hydroxylation events are indicated. The sequences of 203 GAPDH proteins from bacteria and eukaryotes (listed in Supplementary Data File 3) were downloaded from Uniprot (UniProt, 2019) and were aligned using Mega-X software (Kumar, Stecher, Li, Knyaz, & Tamura, 2018). A sequence logo was generated using WebLogo sequence logo generation software (Crooks, Hon, Chandonia, & Brenner, 2004).

Figure S4. TMT mass spectrometric methods. A) Cartoon scheme of the TMT mass spectrometry pipeline. Cultures of *E. coli* or *D. radiodurans* were grown and irradiated as indicated in the *Materials and methods* section. **B)** Example mass spectrum of an unmodified, and hydroxylated peptide.

Figure S5. LFQ mass spectrometric methods. Cartoon scheme of the LFQ mass spectrometry pipeline. MDA-MB-231 cell cultures were prepared and irradiated as indicated in the *Materials and methods* section.

Figure S6. Cartoon scheme of protocol used for whole cell, lysed cells, and dialyzed cell lysate preparation and irradiation. A detailed protocol is described in the *Materials and Methods* section. Briefly, quintuplicate cultures of *E. coli* at early exponential phase growth (OD_{600} of 0.2) was separated into six separate aliquots. Five samples were irradiated with 1,000 Gy. All samples were then lysed via freeze-thaw cycles at -80 °C. Five unirradiated samples were then subjected to 1,000 Gy of IR. Each sample was then dialyzed (3,000 Da molecular weight cutoff) 3X (twice for 2 hr, and once for 16 hr). Five unirradiated samples were then subjected to 1,000 Gy of IR. A portion of all samples was then used for SDS-PAGE or mass spectrometry analysis, as described in the *Materials and Methods*.

Supplemental tables

Supplemental Table 1. Oxidation of the *E. coli* proteome after exposure to 10, 100 or 1,000 Gy.

	0 Gy	10 Gy	100 Gy	1000 Gy
Total peptides detected	6487	5685	6712	6368
Carbonylated (+14 Da) peptides	50	48	50	53
Oxidized (+16 Da) peptides	126	128	158	280
Dioxidized (+32 Da) peptides	35	41	34	46
Trioxidized (+48 Da) peptides	10	1	6	9

Supplemental Dataset Legends

Dataset S1 *E. coli* and *D. radiodurans* pre frac TMT MS data (.xlsx). The datasets produced from tandem mass tag (TMT) mass spectrometry of *E. coli* and *D. radiodurans* treated and untreated with 1,000 Gy of ionizing radiation (IR). Raw datasets, contaminant peptides removed before further analysis, and datasets used for analysis are each listed.

Dataset S2 RAM analysis (.xlsx). Protein abundance values are those determined by Schmidt *et al.* (Schmidt et al., 2016) from *E. coli* at exponential phase growth in growth medium with glycerol as the sole carbon source, similar to the medium used in this study. Proteins listed are mapped from peptides which fit the categories indicated in each sheet.

Dataset S3 GAPDH sequence alignments (.txt). Sequences were downloaded from uniport.org (UniProt, 2019) and were aligned using the MegaX software (Kumar et al., 2018). 203 sequences of glyceraldehyde 3-phosphate dehydrogenase from Bacteria and Eukarya were used for the alignment. Archaeal sequences were excluded, as all archaeal GAPDH active site sequences were divergent from eukaryotic and bacterial sequences.

Dataset S4 MDA-MB-231 cell line LFQ MS data (.xlsx). The dataset produced from label-free quantitation (LFQ) mass spectrometry of human endothelial breast carcinoma MDA-MB-231 cells treated and untreated with 1,000 Gy of ionizing radiation (IR). Raw datasets, and datasets used for analysis are each listed.

Dataset S5 Preliminary *E. coli* MS datasets irradiated with 10, 100 or 1000 Gy(.xlsx). The dataset produced from a preliminary mass spectrometry experiment of single replicate *E. coli* cultures treated with 0, 10, 100, or 1,000 Gy of ionizing radiation (IR). Raw datasets, and datasets used for analysis are each listed.

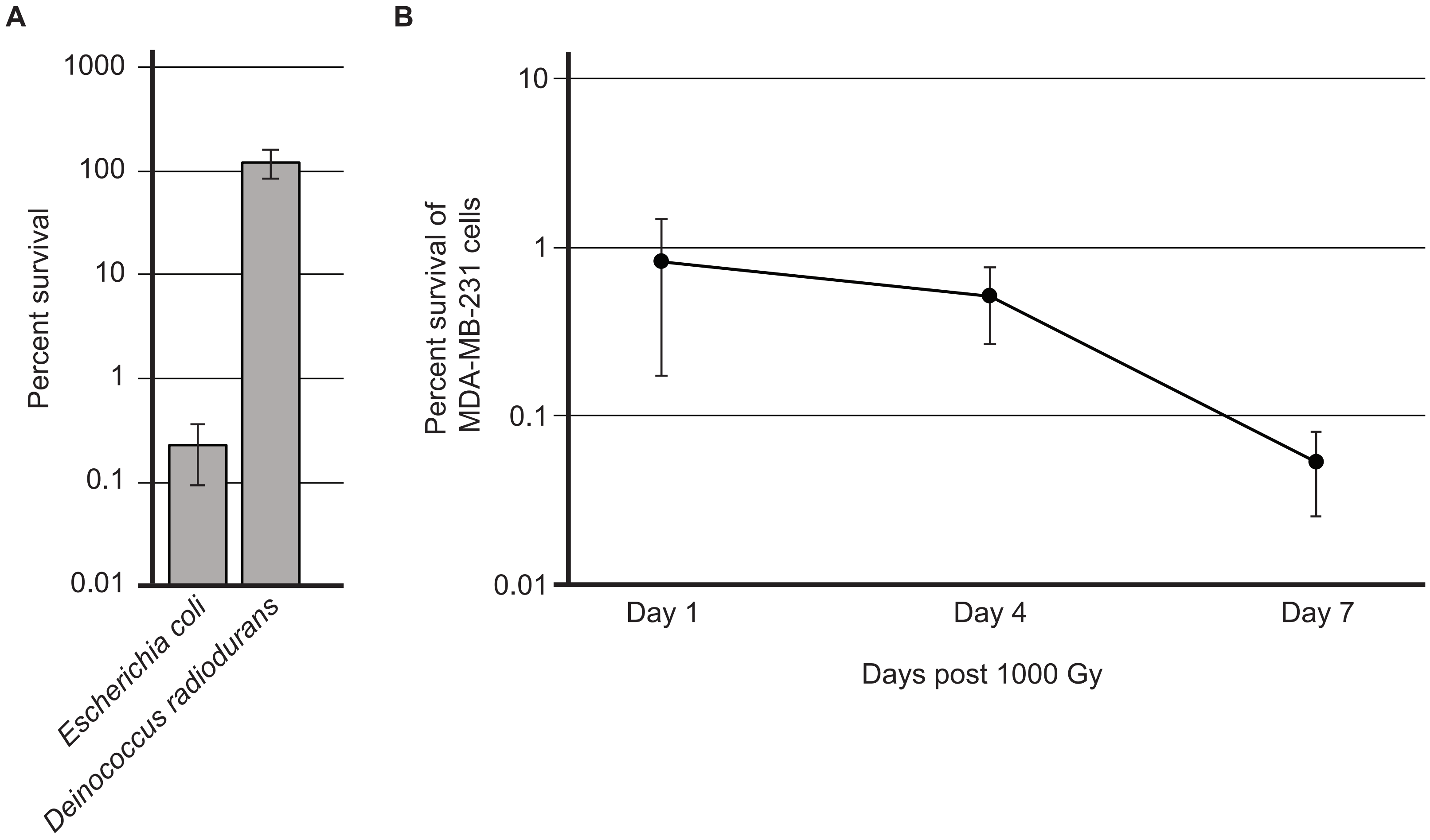
A
B
1000
100
10
1
0.1
0.01
Percent survival
Escherichia coli
Deinococcus radiodurans
10
1
0.1
0.01
Percent survival of
MDA-MB-231 cells
Day 1
Day 4
Day 7
Days post 1000 Gy

Figure S1

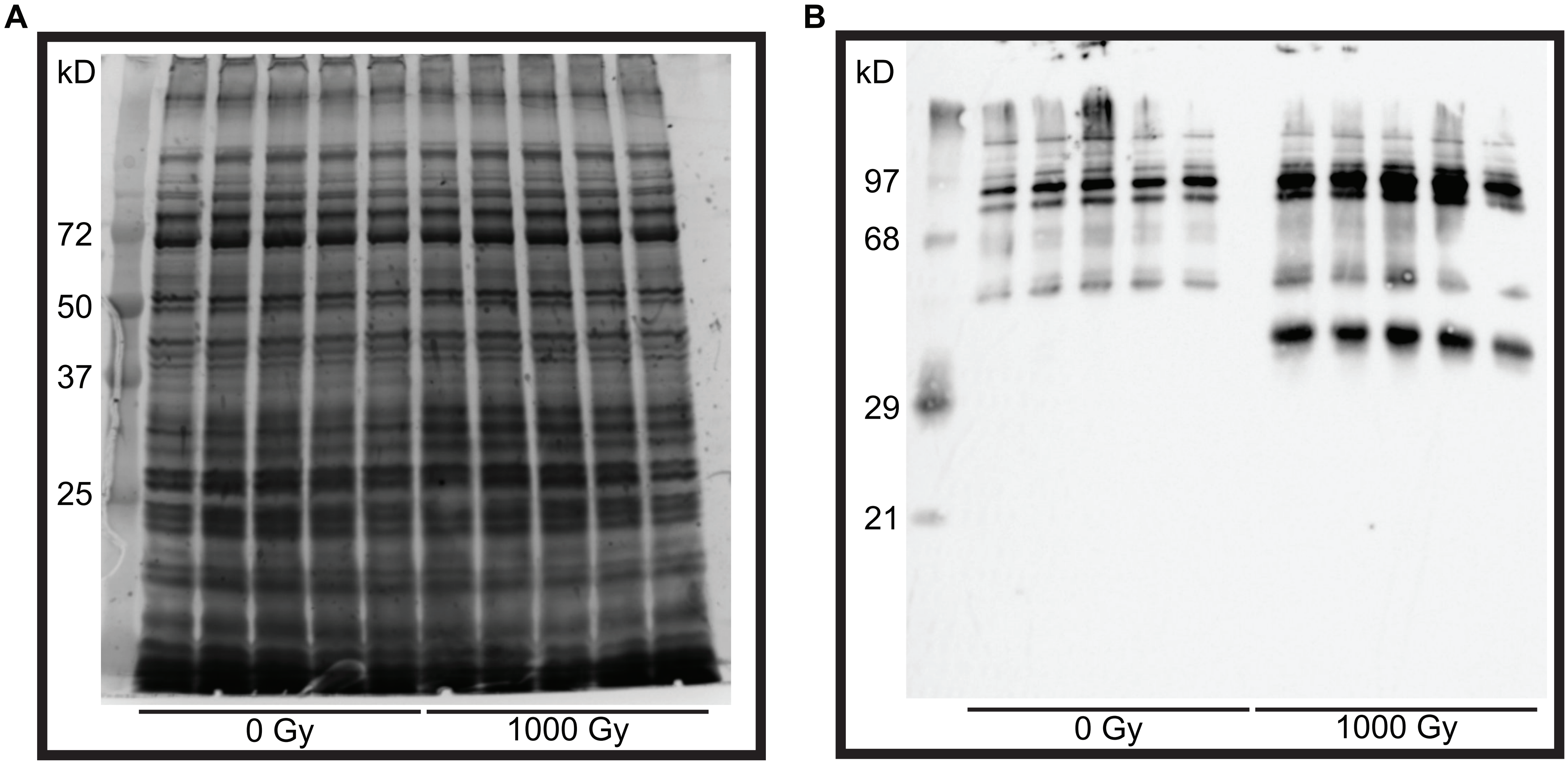

Figure S2

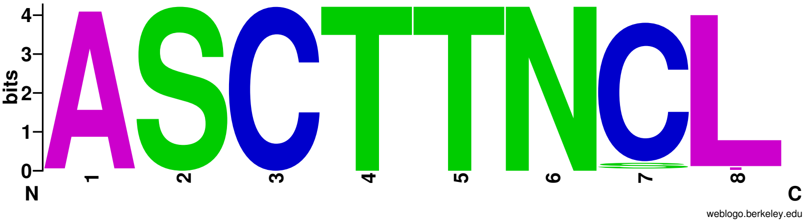

Figure S3

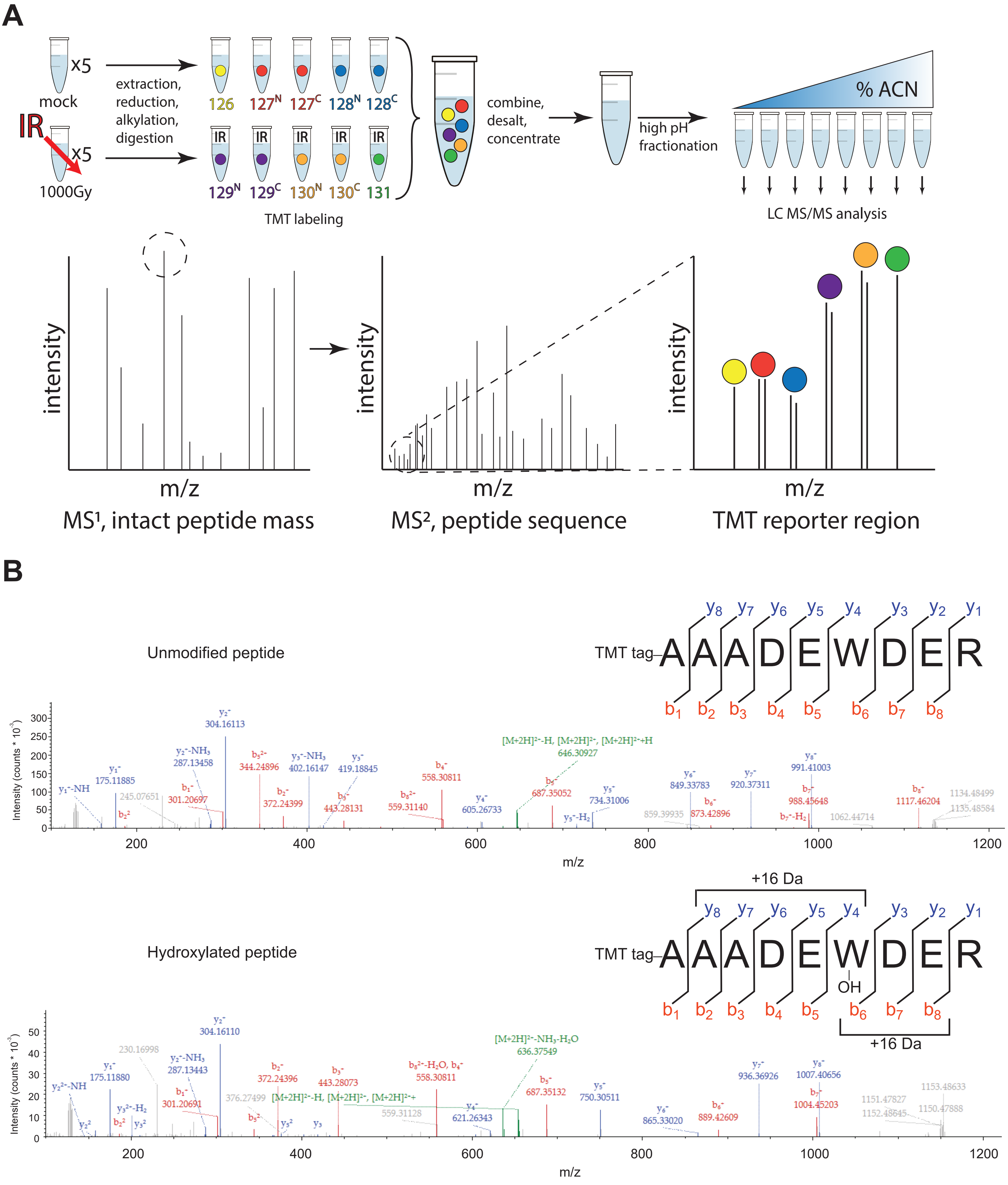
A
mock
x5
IR
1000Gy
x5
extraction, reduction, alkylation, digestion
126
127N
127C
128N
128C
129N
129C
130N
130C
131
TMT labeling
combine, desalt, concentrate
high pH fractionation
% ACN
LC MS/MS analysis
intensity
m/z
MS¹, intact peptide mass
MS², peptide sequence
TMT reporter region
B
Unmodified peptide
TMT tag
AAADEWDER
y8 y7 y6 y5 y4 y3 y2 y1
b1 b2 b3 b4 b5 b6 b7 b8
Intensity (counts * 10^3)
m/z
Hydroxylated peptide
+16 Da
OH

Figure S4

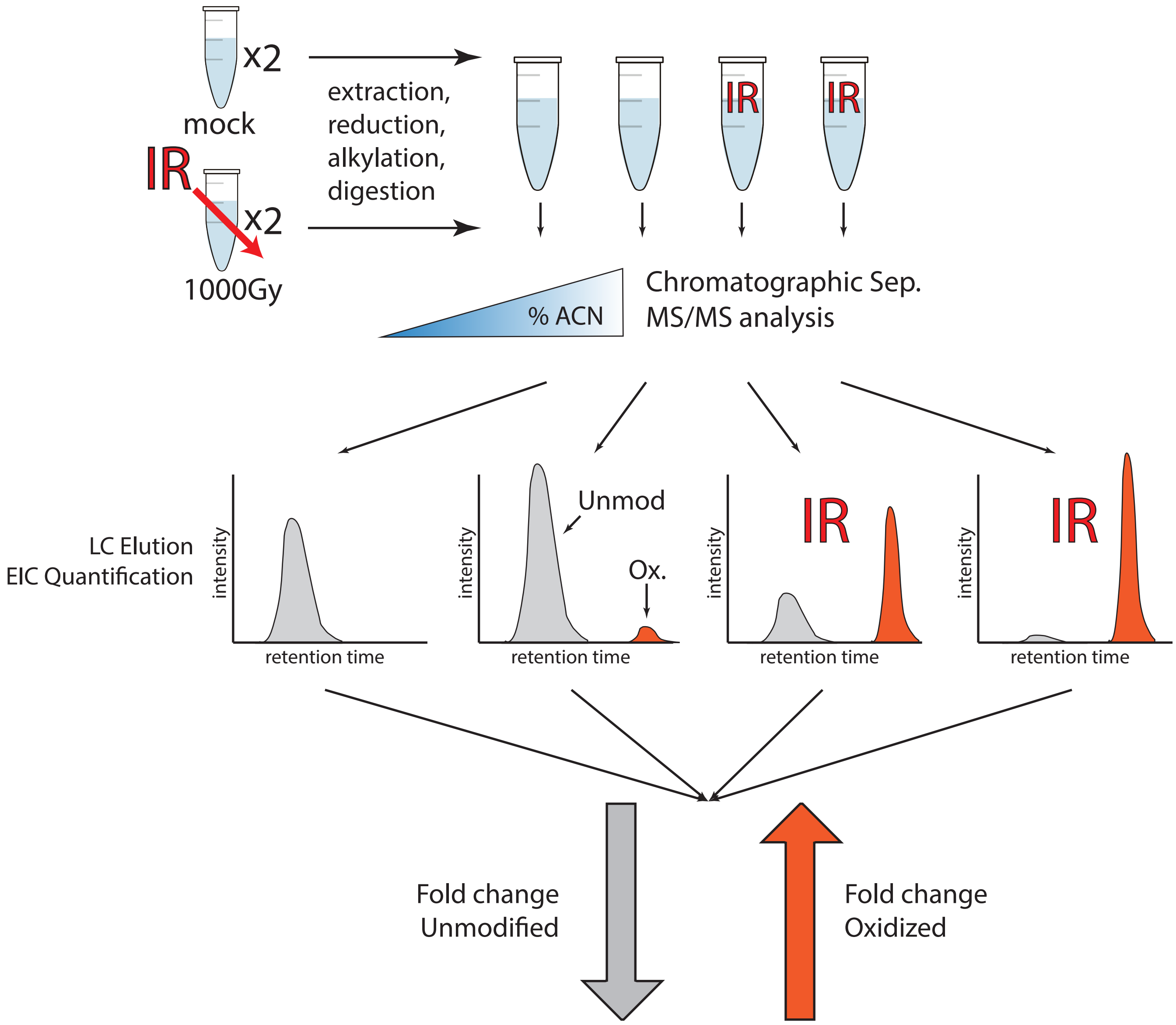
x2
mock
IR
x2
1000Gy
extraction,
reduction,
alkylation,
digestion
IR
IR
% ACN
Chromatographic Sep.
MS/MS analysis
LC Elution
EIC Quantification
intensity
retention time
Unmod
Ox.
IR
IR
Fold change
Unmodified
Fold change
Oxidized

Figure S5

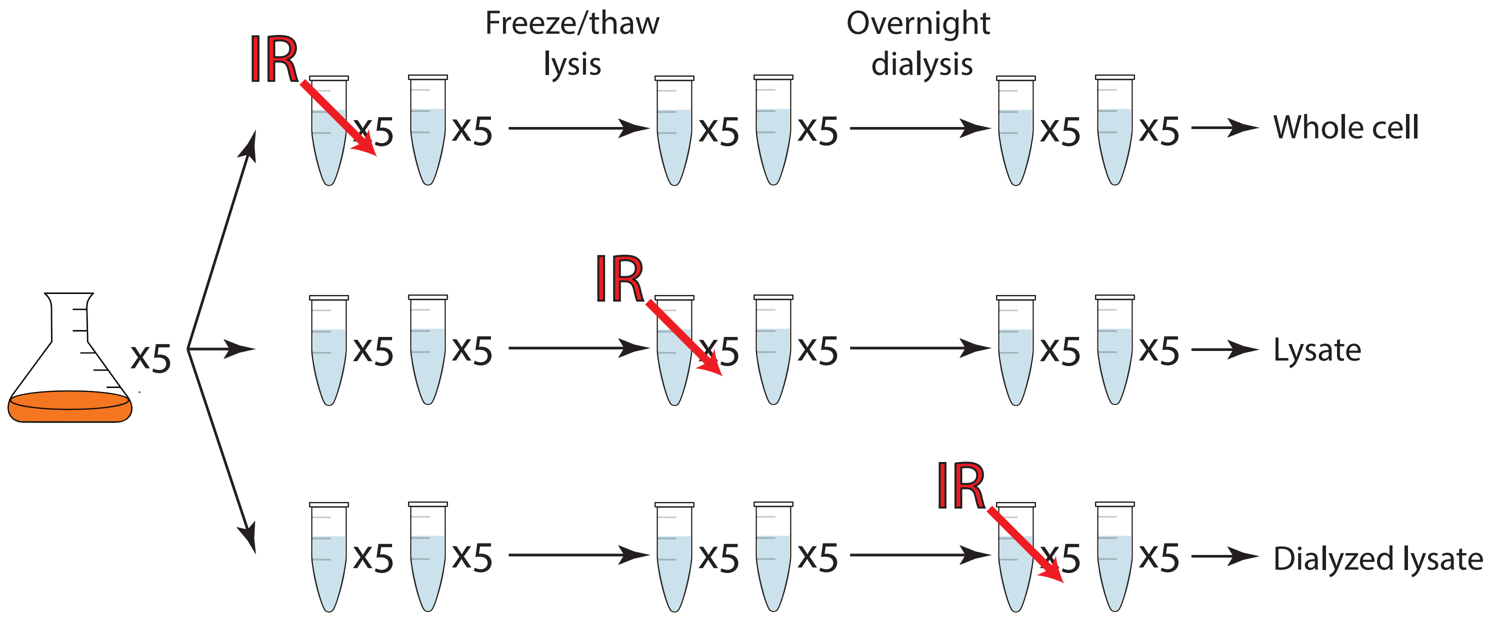
x5
IR
Freeze/thaw lysis
Overnight dialysis
x5
Whole cell
Lysate
Dialyzed lysate

Figure S6
